# Inhibition of the renin-angiotensin system causes concentric hypertrophy of renal arterioles in mice and humans

**DOI:** 10.1172/jci.insight.154337

**Published:** 2021-12-22

**Authors:** Hirofumi Watanabe, Alexandre G. Martini, Evan A. Brown, Xiuyin Liang, Silvia Medrano, Shin Goto, Ichiei Narita, Lois J. Arend, Maria Luisa S. Sequeira-Lopez, R. Ariel Gomez

**Affiliations:** 1 Department of Pediatrics, Child Health Research Center, University of Virginia School of Medicine, Charlottesville, Virginia, USA.; 2 Division of Clinical Nephrology and Rheumatology, Kidney Research Center, Niigata University Graduate School of Medical and Dental Sciences, Niigata, Niigata, Japan.; 3 Department of Pathology, Johns Hopkins University and Johns Hopkins Hospital, Baltimore, Maryland, USA.

**Keywords:** Nephrology, Vascular Biology, Chronic kidney disease, Homeostasis, Hypertension

## Abstract

Inhibitors of the renin-angiotensin system (RAS) are widely used to treat hypertension. Using mice harboring fluorescent cell lineage tracers, single-cell RNA-Seq, and long-term inhibition of RAS in both mice and humans, we found that deletion of renin or inhibition of the RAS leads to concentric thickening of the intrarenal arteries and arterioles. This severe disease was caused by the multiclonal expansion and transformation of renin cells from a classical endocrine phenotype to a matrix-secretory phenotype: the cells surrounded the vessel walls and induced the accumulation of adjacent smooth muscle cells and extracellular matrix, resulting in blood flow obstruction, focal ischemia, and fibrosis. Ablation of the renin cells via conditional deletion of **β**_1_ integrin prevented arteriolar hypertrophy, indicating that renin cells are responsible for vascular disease. Given these findings, prospective morphological studies in humans are necessary to determine the extent of renal vascular damage caused by the widespread use of inhibitors of the RAS.

## Graphical abstract

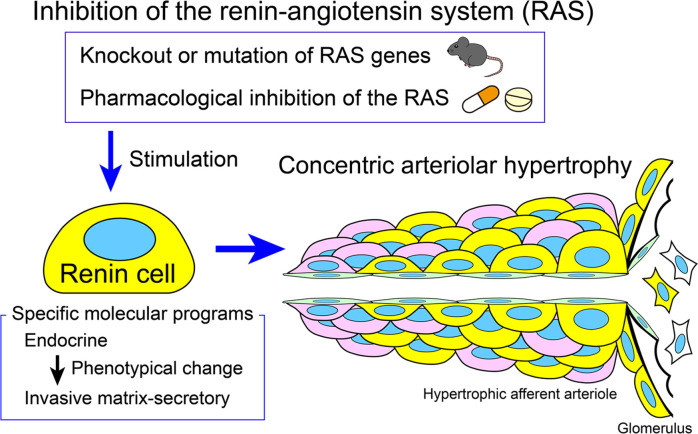

## Introduction

Hypertension affects more than 1 billion people worldwide (1) and is a major risk factor for cardiovascular disease, stroke, end-stage renal disease, and death (2). Vascular hypertrophy with attendant organ injury is a serious disorder usually associated with severe, chronic hypertension (3). Interestingly, deletion of the renin-angiotensin system (RAS) genes — usually associated with hypotension — leads to an arterial phenotype indistinguishable from that observed in uncontrolled hypertension (4). How 2 conditions with opposite blood pressure levels can lead to a similar arterial phenotype is puzzling. In neither condition, the underlying events leading to the disease are clear.

We have noted that experimental or spontaneous mutations of the RAS genes or treatment with renin-angiotensin inhibitors (RASi) in mammals results in increased cells programmed for the renin phenotype along and within the walls of the renal arterioles (4–13). Those findings suggested that renin cells were involved in the pathogenesis of vascular disease. Using genetically modified mice harboring fluorescent cell lineage tracers, single-cell RNA-Seq, and long-term inhibition of RAS in both mice and humans, we tested (a) whether renin cells were directly involved in the arterial disease, (b) whether renin cells changed their identity and were transformed during the evolution of the arterial pathology, (c) whether renin cells synthesized factors that modify the composition of the extracellular milieu responsible for the arterial disease, and (d) whether a similar arterial disease was observed in humans treated with RASi.

## Results

### Ablation of the renin gene results in multiclonal concentric arteriolar hypertrophy.

**Figure 1:**
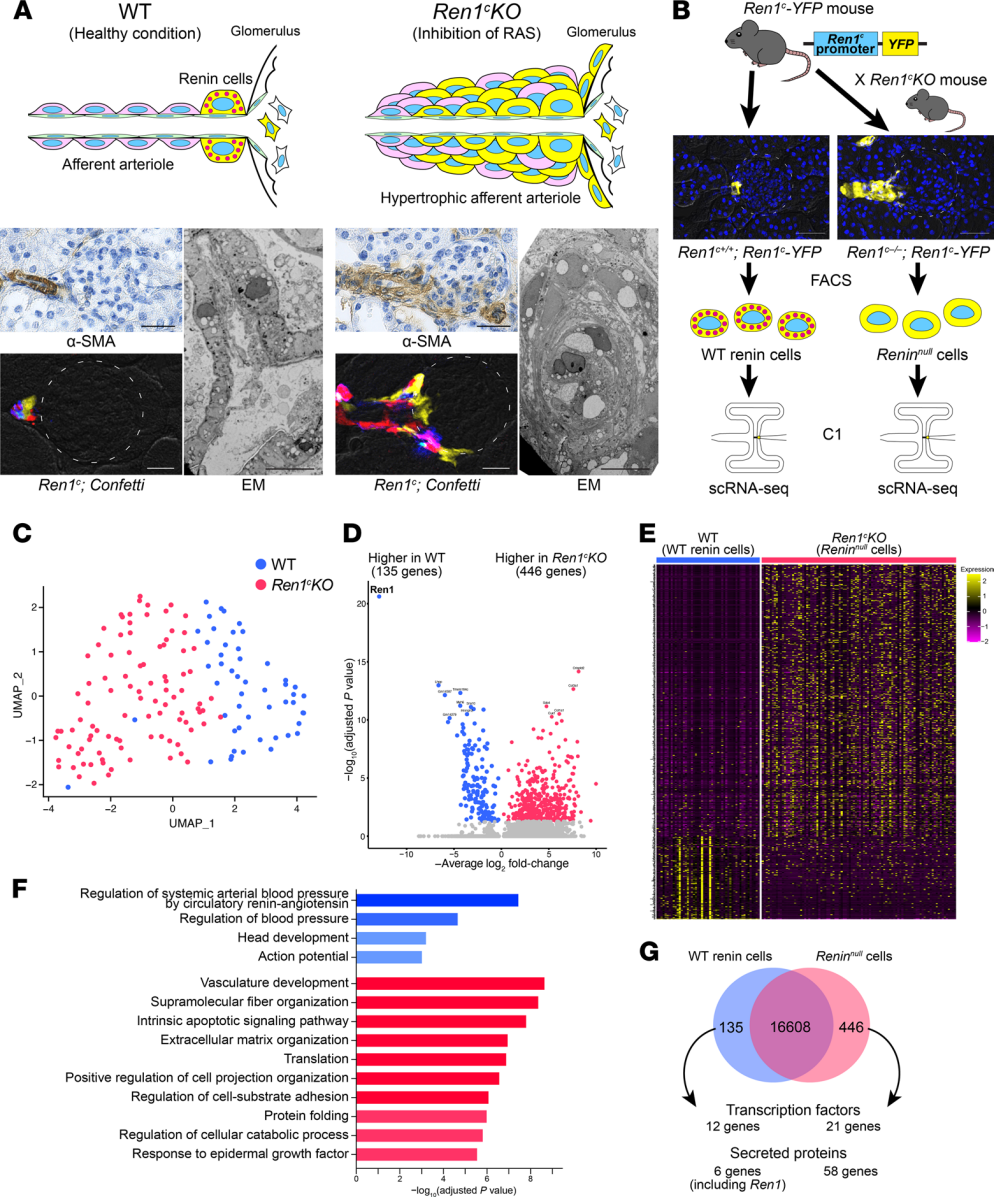
*Renin^null^* cells have different transcriptomic profiles. (**A**) Pathological findings of *Ren1^c^-KO* mice. Immunohistochemistry for α-SMA showed hypertrophic arterioles in *Ren1^c^-KO* mouse kidneys. Scale bars: 20 μm. Kidney sections from *Ren1^c^-KO Ren1^c^-Cre Confetti* mice showed multiple colors in the hypertrophic arterioles, suggesting the multiclonality of *Renin^null^* cells. Scale bars: 20 μm. Left: WT mice showed normal architecture of periglomerular arterioles with a single layer of smooth muscle cells (SMCs). Right: *Ren1^c^-KO* mice showed concentric hypertrophy of arteriolar smooth muscle. Scale bars: 10 μm. (**B**) Schematic of scRNA-Seq of WT renin cells and *Renin^null^* cells. *Ren1^c^-YFP* mice have a transgene consisting of the *YFP* genes driven by the *Ren1^c^* gene regulatory region. YFP-positive kidney cells from *Ren1^c+/+^ Ren1^c^-YFP* mice and *Ren1^c–/–^ Ren1^c^-YFP* mice were isolated by FACS. Single cells were captured with the Fluidigm C1 System, and scRNA-Seq was performed. Scale bars: 50 μm. (**C**) The UMAP with all the cells after normalization. (**D**) Volcano plot showing the differentially expressed genes between WT renin cells and *Renin^null^* cells. (**E**) Heatmap analysis with differentially expressed genes showed a clear separation of WT renin cells and *Renin^null^* cells. (**F**) The 10 most enriched categories identified by GO analysis on genes higher in WT renin cells (red) and genes higher in *Renin^null^* cells (blue). (**G**) Venn diagram showing differentially expressed genes. α-SMA, α–smooth muscle actin; EM, electron microscopy; FACS, fluorescence-activated cell sorting; GO, Gene Ontology; *Ren1^c^-KO*, *Ren1^c^* gene knockout; scRNA-Seq, single-cell RNA sequencing; UMAP, uniform manifold approximation and projection; WT, wild-type.

Deletion of the renin gene led to progressive thickening of the afferent arterioles and interlobular arteries (Figure 1A). The arterial thickening was caused by an increase in the number of *Renin^null^* cells and by the inward accumulation of SMCs (Figure 1A, right, top, and bottom panels). Immunohistochemistry for α-SMA and EM of the kidneys demonstrated that the increase in the number of cells and consequent thickening of the afferent arterioles and interlobular arteries resulted in a marked reduction in the arteriolar lumen (Figure 1A, right bottom). To trace the fate of *Renin^null^* cells during the progression of the arteriolar hypertrophy and define whether cells of the renin lineage expand in a monoclonal or multiclonal fashion, we generated *Ren1^c–/–^ Ren1^c^-Cre R26.Confetti* (*Ren1^c^-KO*) and *Ren1^c+/–^ Ren1^c^-Cre R26.Confetti* (control) mice and studied them at 2, 4, and 6 months of age. In these mice, the Cre recombinase is expressed under the regulation of the *Ren1^c^* gene promoter. As the mice got older, the thickness of the afferent arterioles and the surrounding kidney fibrosis worsened (Supplemental Figure 1; supplemental material available online with this article; https://doi.org/10.1172/jci.insight.154337DS1). In mice with an intact *Ren1^c^* gene, the juxtaglomerular (JG) cells were of multiple individual colors, suggesting they originated from multiple clones. In the *Ren1^c^-KO* mice, this mosaic multiclonal pattern was retained along the arterioles even after 6 months of disease progression and severe arteriolar hypertrophy (Figure 1A and Supplemental Figure 1). This indicates that there was no preference of one clone of cells over others in the maintenance and progression of the arteriolar disease. As we previously showed using a different reporter mouse (9), the cells from the renin lineage also contributed to the periglomerular fibrosis. This is consistent with a recent publication corroborating our previous findings (14). EM images showed a disorganized appearance of preglomerular arterioles in *Ren1^c^-KO* mice, with a marked layering of SMCs and increased basement membrane material (Figure 1A, right bottom) compared with the single organized smooth muscle layer found in WT mice (Figure 1A, left bottom). Thus, deletion of the renin gene resulted in the concentric accumulation of multiclonal *Renin^null^* cells, causing the concentric hypertrophy of the afferent and interlobular arterioles.

### Renin^null^ cells develop a matrix-secretory phenotype.

To define whether deletion of the renin gene altered the transcriptional identity of renin cells, we performed scRNA-Seq in cells sorted from mice with an intact renin gene (WT mice: *Ren1^c+/+^ Ren1^c^-YFP,* WT renin cells) and from mice with deletion of the renin gene (*Ren1^c^-KO* mice: *Ren1^c–/–^ Ren1^c^-YFP*, *Renin^null^* cells) (Figure 1B). Both mice harbored a *Ren1^c^-YFP* transgene where expression of YFP is driven by the 5′ regulatory sequence of the *Ren1^c^* gene (15). Cells were isolated by FACS as previously described (16, 17) and subjected to scRNA-Seq using the Fluidigm C1 System (Figure 1B). The transcriptional landscape of *Renin^null^* cells was markedly different from the profile of WT renin cells (Figure 1, C–G). This was reflected in the clear separation between WT renin cells and *Renin^null^* cells, which formed 2 different clusters (UMAP, Figure 1C). As expected, cells in both groups showed detectable expression of *Akr1b7,* a previously established renin cell marker gene (refs. 18, 19; and Supplemental Figure 2, A and B). This was confirmed by in situ hybridization (ISH; Supplemental Figure 2C).

Further, the differential analysis identified 581 genes that were expressed differently between WT renin cells and *Renin^null^* cells (Figure 1, D and E, and Supplemental Table 1). Differences in the volcano plot and heatmap were reflected in the GO analysis related to the blood pressure regulation in WT renin cells, whereas *Renin^null^* cells’ GO terms were related to vascular development, fiber and matrix organization, and apoptotic pathways (Figure 1F), in correspondence with the phenotypic transformation of *Renin^null^* cells and the morphological appearance of the arterial disease. Interestingly, genes expressed by activated myofibroblasts and vascular SMCs (*Acta2*, *Tagln*, *Cnn1,* and *Myh11*) were highly detected in the *Renin^null^* cells (Supplemental Figure 2D), indicating that the *Renin^null^* cells, while attempting to maintain an endocrine phenotype, also transform to a myofibroblastic/fibrotic phenotype in contradistinction with the WT renin cells.

Out of the 581 differentially expressed genes (DEGs) between WT renin cells and *Renin^null^* cells, we identified 33 transcription factor (TF) genes and 64 secreted protein genes (Figure 1G). We, therefore, further analyzed the function of these DEGs in *Renin^null^* cells.

**Figure 2:**
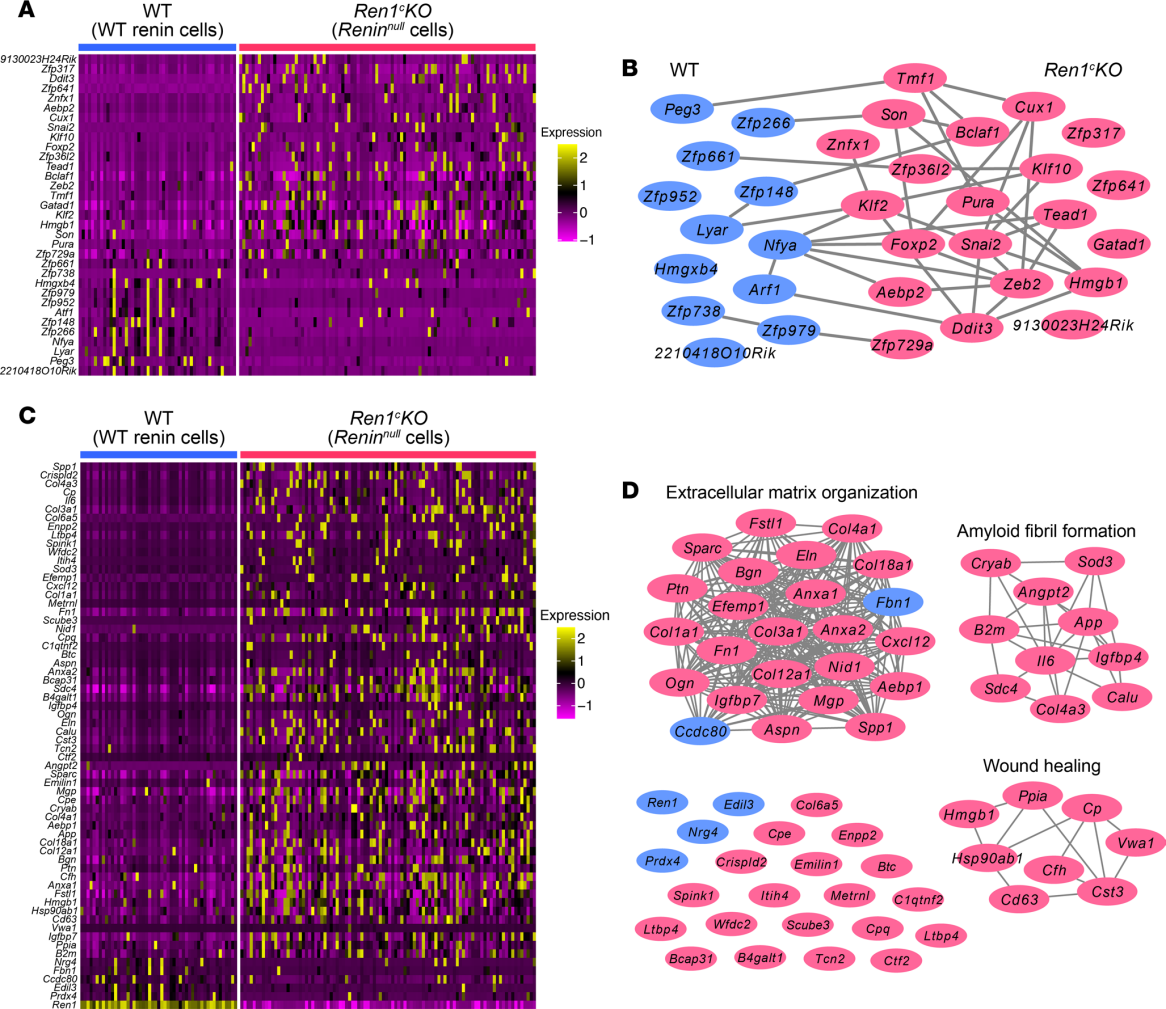
scRNA-Seq of *Renin^null^* cells identifies specific TFs and secreted proteins. (**A**) Heatmap of the TF genes differentially expressed between WT renin cells and *Renin^null^* cells. (**B**) PPI enrichment analysis using differentially expressed TF genes. The genes higher in WT renin cells and those higher in *Renin^null^* cells are shown in blue and red, respectively. (**C**) Heatmap of the secreted protein genes differentially expressed between WT renin cells and *Renin^null^* cells. (**D**) PPI enrichment analysis using differentially expressed secreted protein genes. The genes higher in WT renin cells and those higher in *Renin^null^* cells are shown in blue and red, respectively. PPI, protein-protein interaction; scRNA-Seq, single-cell RNA sequencing; WT, wild-type.

To define which TFs and/or secreted proteins may be involved in the phenotypic transformation of *Renin^null^* cells and responsible for their effect on nearby SMCs, we compared the transcriptional profile of *Renin^null^* versus WT cells. Of the 33 differentially expressed TFs, 12 were upregulated in the WT renin cells, and 21 were upregulated in *Renin^null^* cells (Figure 1G and Supplemental Table 2). These TFs separated the WT and *Renin^null^* cells clearly (Figure 2A). Using the protein-protein interaction (PPI) enrichment analysis, we found that the TFs upregulated in the *Renin^null^* cells were clustered together more than those in WT renin cells (Figure 2B) and related to the regulation of DNA-templated transcription in response to stress (*Ddit3,* *Klf2,* *Bclaf1,* etc.). For instance, DNA-damage inducible transcript 3 (*Ddit3*) regulates endoplasmic reticulum (ER) stress–induced apoptosis (20). Krüppel-like factor 2 (*Klf2*) has been reported to be involved in the inhibition of cell proliferation (21). In fact, we recently showed that under conditions of constant stimulation, renin cells do not proliferate (22). In aggregate, the TFs overexpressed by the null cells might be due to their transformation to an invasive phenotype and the production of molecules that lead to the concentric arterial hypertrophy by influencing the behavior of nearby cells.

**Figure 3:**
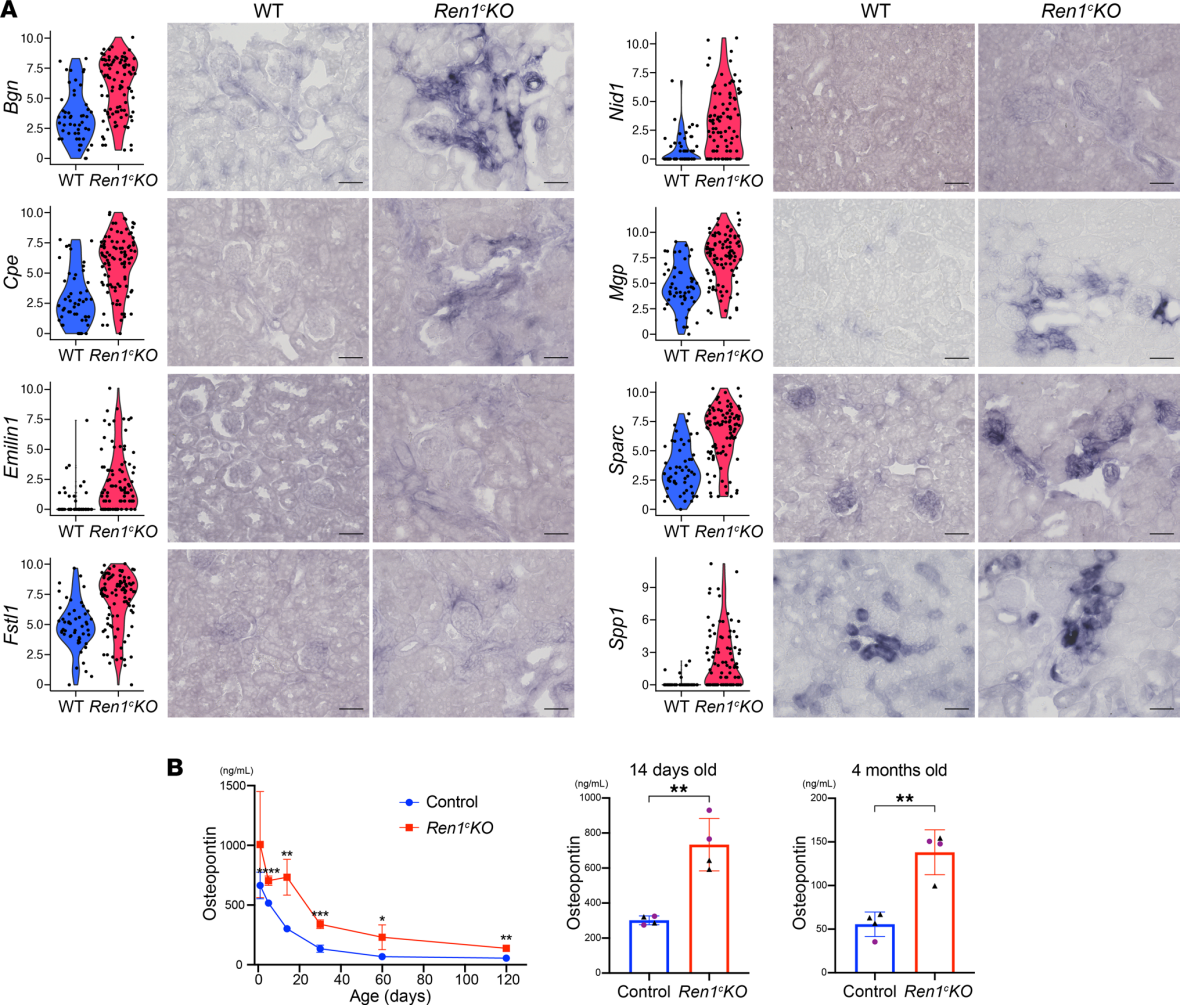
*Renin^null^* cells secrete proteins underlying the vascular abnormalities. (**A**) Violin plots and validation by ISH of selected secreted protein genes upregulated in the *Renin^null^* cells. The expression of the genes was detected in the hypertrophic arterioles at higher levels in *Ren1^c^-KO* than WT. Scale bars: 50 μm. (**B**) ELISA for osteopontin during disease development (*n* = 4, Student’s *t* test). All data are reported as means ± standard deviation. *P < 0.05, **P < 0.01, ***P < 0.001, ****P < 0.0001. ISH in situ hybridization, *Ren1^c^-KO*, *Ren1^c^* gene knockout; scRNA-Seq, single-cell RNA sequencing; WT, wild-type.

In fact, we found that *Renin^null^* cells expressed a large number of significant genes (58 genes) encoding secreted proteins versus only 6 genes (including *Ren1*) expressed in WT renin cells (Figure 2C and Supplemental Table 3). These secreted protein genes also clearly separated the WT renin cells from the *Renin^null^* cells (Figure 2C). The PPI enrichment analysis identified extracellular matrix organization, amyloid fibril formation, and wound healing as the best 3 scoring GO terms for these differentially expressed secreted protein genes (Figure 2D). These results indicate that *Renin^null^* cells affected the composition of their extracellular environment and the behavior and fate of the cells nearby. Using ISH, we confirmed that those secreted protein genes were upregulated in the *Renin^null^* cells. The expression of *Bgn,* *Cpe,* *Emillin1,* *Fstl1,* *Nid1,* *Mgp,* *Sparc,* and *Spp1* was increased in the hypertrophic arterioles in *Ren1^c^-KO* mice compared with the afferent arterioles in WT mice (Figure 3A). Secreted protein acidic and rich in cysteine (*Sparc*) modulates cell-matrix interactions and cell function (23). Osteopontin (*Spp1*) plays a role in the renal damage associated with inflammatory glomerulonephritis, obstructive uropathy, and tubule interstitial disease (24). Biglycan (*Bgn*) was reported to be increased in several types of renal diseases (25). We hypothesized that these secreted protein genes could be biomarkers of renal diseases with arteriolar abnormality. ELISA of plasma samples at several ages showed increased levels of osteopontin at every time point of *Ren1^c^-KO* mice compared with the control (Figure 3B). Intriguingly, even at P5, when the vascular abnormality was not yet obvious in the kidney tissue (Supplemental Figure 3), the plasma osteopontin was significantly higher in *Ren1^c^-KO* mice, suggesting that osteopontin could be used as a predictive marker of renal arteriolar hypertrophy.

### Renin^null^ mural cells cluster into cells that attempt to make renin and SMCs that do not.

**Figure 4:**
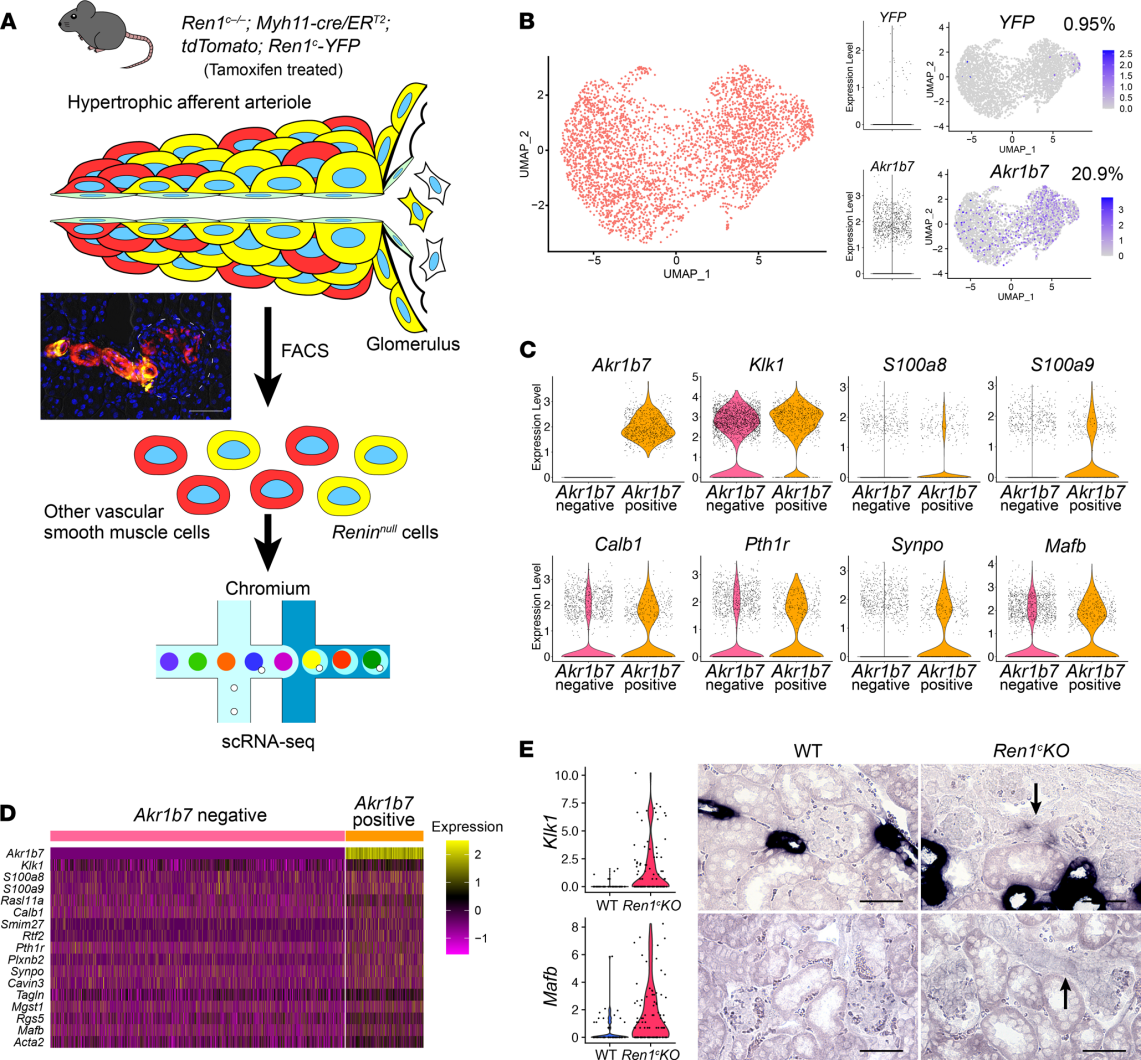
*Renin^null^* mural cells comprise 2 phenotypically different groups of cells. (**A**) Schematic of scRNA-Seq of *Renin^null^* cells and other vascular SMCs. Fluorescent cells from *Ren1^c–/–^ Myh11-CreER^T2^ tdTomato Ren1^c^-YFP* mouse kidneys were isolated by FACS. scRNA-Seq was performed with the 10x Genomics Chromium System. (**B**) The UMAP with all the cells after normalization and volcano plots and feature plots for *YFP* and *Akr1b7*. Respectively, 0.95% and 20.9% of the cells were positive for *YFP* and *Akr1b7*. (**C**) Violin plots of representative DEGs between *Akr1b7*-negative cells and *Akr1b7*-positive cells. (**D**) Heatmap analysis with DEGs between *Akr1b7*-negative cells and *Akr1b7*-positive cells. (**E**) Violin plots from C1 scRNA-Seq and ISH for *Klk1* and *Mafb* mRNA in WT and the *Ren1^c^-KO* kidneys. Arrows indicate hypertrophic afferent arterioles. Scale bars: 50 μm. DEGs, differentially expressed genes; FACS, fluorescence-activated cell sorting; GO, Gene Ontology; ISH, in situ hybridization; *Mafb*, v-maf musculoaponeurotic fibrosarcoma oncogene family, protein B; *Ren1^c^-KO*, *Ren1^c^* gene knockout; scRNA-Seq, single-cell RNA sequencing; SMCs, smooth muscle cells; UMAP, uniform manifold approximation and projection; WT, wild-type.

**Figure 5:**
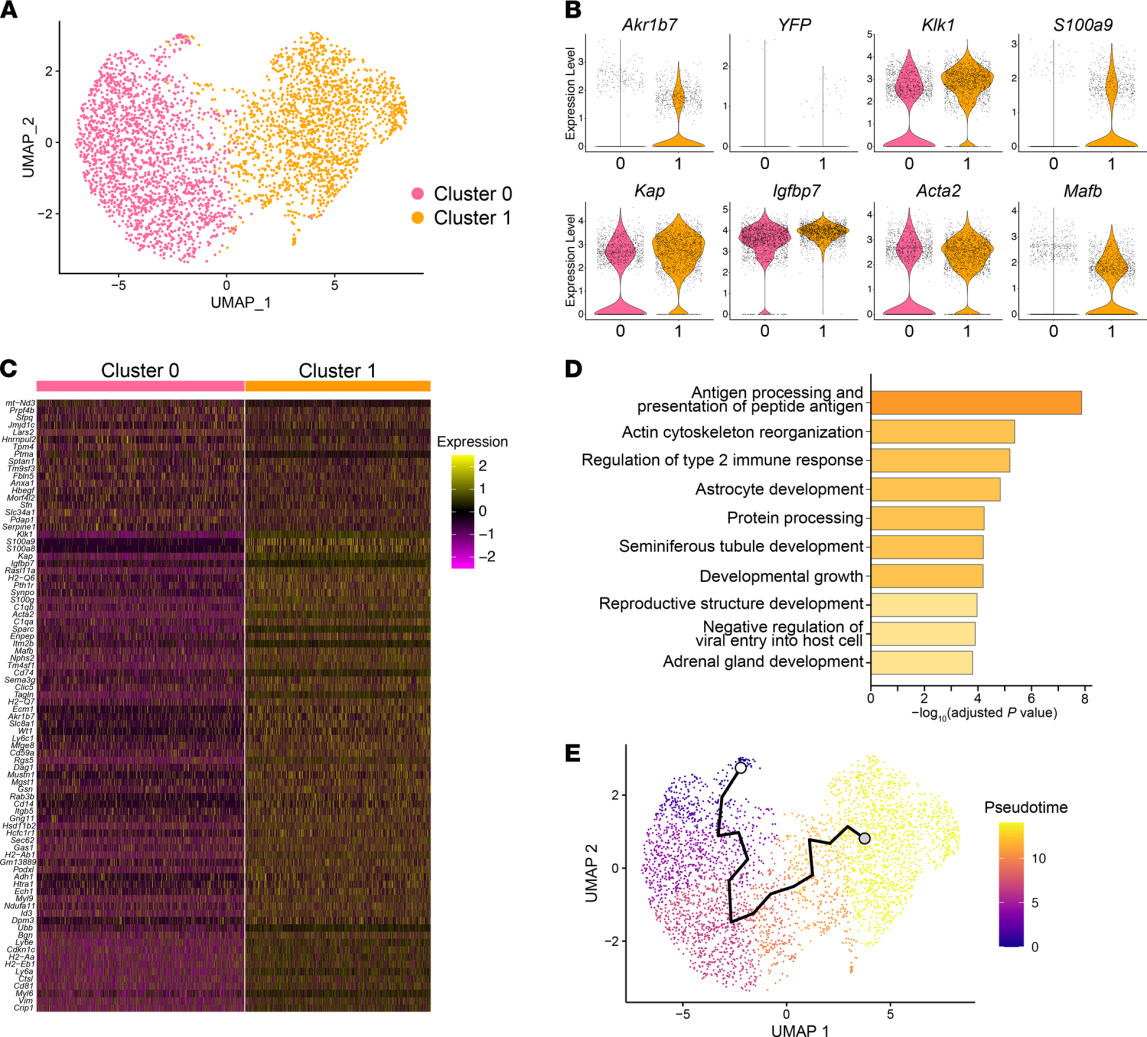
Phenotypical transition from SMCs to endocrine *Renin^null^* cells. (**A**) UMAP with unbiased clustering indicated 2 clusters in scRNA-Seq of *Renin^null^* cells and other vascular SMCs. (**B**) Violin plots of representative DEGs between clusters 0 and 1. (**C**) Heatmap analysis with DEGs between clusters 0 and 1. (**D**) The 10 most enriched categories identified by GO analysis on DEGs between clusters 0 and 1. (**E**) Unbiased trajectory analysis of cells of *Ren1^c^-KO* mice. DEGs, differentially expressed genes; FACS, fluorescence-activated cell sorting; GO, Gene Ontology; ISH, in situ hybridization; scRNA-Seq, single-cell RNA sequencing; SMCs, smooth muscle cells; UMAP, uniform manifold approximation and projection; WT, wild-type.

To further investigate the differences between *Renin^null^* cells and surrounding vascular SMCs, we generated *Ren1^c–/–^ Myh11-CreER^T2^ tdTomato Ren1^c^-YFP* mice (Figure 4A). By injecting tamoxifen at adult ages, we labeled vascular SMCs with tdTomato. This approach also resulted in the labeling of *Renin^null^* cells, which also synthesized smooth muscle proteins but could be distinguished by their dual expression of red and yellow fluorescent proteins, whereas other vascular SMCs not destined to the renin phenotype were only red (Figure 4A and Supplemental Figure 4A). Kidney cortices from 2 male mice were dissociated into single cells. Cells were sorted and collected according to their red or yellow colors (Figure 4A and Supplemental Figure 4B). Of the cells we collected, 19% were YFP positive, and 81% were YFP negative but tdTomato positive. Next, we performed scRNA-Seq using the 10x Genomics Chromium System (Figure 4A). After generating cDNA libraries, sequencing, and quality control, we obtained the transcriptional profile of 3594 cells. The UMAP of Figure 4B shows the distribution of these cells according to their expression profile. Because mRNA expression of fluorescent reporters does not correspond to their protein expression (26), we used *Akr1b7,* a typical marker for renin cells (independently of the expression of renin; refs. 9, 18), and compared the cells that were positive with the cells that were negative for *Akr1b7*. The percentage of *Akr1b7*-positive cells in this scRNA-Seq (20.9%) was similar to that of the FACS result for YFP (Figure 4B and Supplemental Figure 4B). Then, we compared the transcriptomic profiles of *Akr1b7*-positive and -negative cells and detected 17 DEGs, including *Akr1b7* (Figure 4, C and D, and Supplemental Table 4), all of which were upregulated in the *Akr1b7*-positive cells. A prominent DEG was *Klk1* (kallikrein 1), a major component of the kallikrein-kinin system that crosstalks with the RAS (27). ISH showed that *Klk1* was not only expressed in collecting ducts, as expected, but also expressed in the thickened arterioles of the *Ren1^c^-KO* mice (Figure 4E, top panels). *Mafb* is a TF that plays a role in the development of the hindbrain structures, the thymus, interneurons, pancreatic islet cells, and the hematopoietic system (28). By ISH, *Mafb* was ubiquitously expressed in the kidney, including *Renin^null^* cells (Figure 4E, bottom panels). The role(s) of *Klk1* and *Mafb* in the phenotypic transformation of *Renin^null^* cells and the arterial disease remains to be determined. We further analyzed this scRNA-Seq data with an unbiased hierarchical clustering method, and the cells were separated into 2 clusters, cluster 0 and cluster 1 (Figure 5A). With differentially expressed analysis, we found 84 DEGs between cluster 0 and 1, of which 18 and 66 genes were upregulated in cluster 0 and 1, respectively (Figure 5, B and C, and Supplemental Table 5). *Akr1b7* was 1 of the cluster 1–upregulated genes. In cluster 1, 32.9% of the cells were positive for *Akr1b7*, whereas in cluster 0, only 10.2% of cells were positive for *Akr1b7*. *Klk1* had the lowest adjusted *P* value of all the DEGs that were upregulated in cluster 1. These results suggest that cluster 1 had more *Renin^null^* cell–like phenotypes. To investigate the differences between *Renin^null^* cells and other vascular SMCs, we performed GO enrichment analysis of biological processes between cluster 0 and 1 (Figure 5D). We detected GO terms related to immune response and cell development. These findings suggest that *Renin^null^* cells had specific transcriptional programs that may be involved in the vascular abnormalities of the afferent arterioles in *Ren1^c^-KO* mice. To support these findings, we constructed a trajectory analysis using the unbiased UMAP (Figure 5E). *Akr1b7* was expressed along the trajectory (Figure 4B and Figure 5E). This trajectory was composed of genes that we detected in the *Akr1b7*-positive cells (i.e., *Klk1*, *Mafb*; Supplemental Table 6), suggesting that there was a phenotypical transition from SMCs to *Renin^null^* cells.

The cAMP pathway is known to be important for the regulation and maintenance of renin cells (15–17). We found that the expression of the genes related to the cAMP pathway was maintained in *Renin^null^* cells (Supplemental Figure 5, A and B) and increased in the *Renin^null^* cells when compared with the surrounding SMCs (Supplemental Figure 5, C and D). Recently, we reported that renin cells possess a nuclear mechanotransduction pressure-sensing mechanism, the renal baroreceptor, that regulates renin gene expression (16). Our scRNA-Seq showed that the genes encoding the proteins that constitute the baroreceptor mechanism, particularly *Itgb1* and *Lmna*, were increased within the trajectory toward the renin phenotype (Supplemental Figure 5E). These results indicate that *Renin^null^* cells are continuously transformed in their futile attempt to achieve a renin cell–like identity, even though it is impossible for them to generate renin.

Taken together, *Renin^null^* cells not only were acting as a major cellular component of the hypertrophic arterioles but also showed a specific transcriptional profile that may be related to both the cause and consequence of the progression of the vascular disease.

### Lack of integrin β_1_ in the Renin^null^ cells ameliorates the afferent arteriolar hypertrophy.

**Figure 6:**
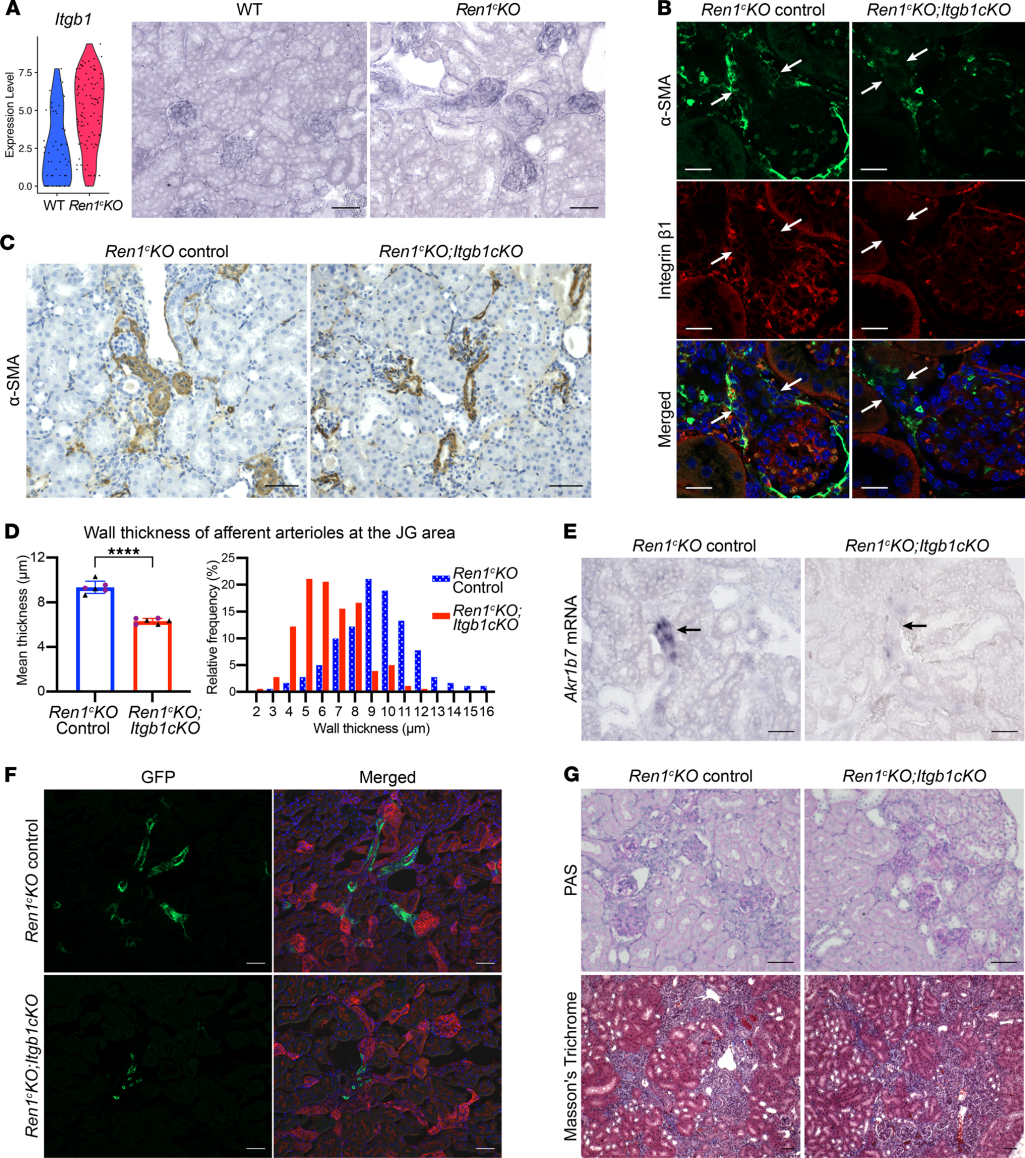
Deletion of integrin β_1_ in renin lineage cells inhibits arteriolar hypertrophy in *Ren1^c^-KO* mice. (**A**) Violin plots and ISH for *Itgb1* mRNA in *Renin^null^* cells. Scale bars: 50 μm. (**B**) Immunofluorescence staining for α-SMA and integrin β1 in kidneys from *Ren1^c^-KO* control and *Ren1^c^-KO* with *Itgb1-cKO* mice. Arrows indicate the α-SMA–positive cells at the JG area. Integrin β_1_ was not observed at the JG area of the *Ren1^c^-KO Itgb1-cKO* mice. Scale bars: 20 μm. (**C** and **D**) Arteriolar hypertrophy was prevented in *Ren1^c^-KO Itgb1-cKO* mice. Immunohistochemistry for α-SMA showed decreased thickness of the walls of afferent arterioles in *Ren1^c^-KO Itgb1-cKO* mice compared with *Ren1^c^-KO* control (**C**). Scale bars: 50 μm. The mean wall thickness of afferent arterioles at the JG area was significantly smaller in *Ren1^c^-KO Itgb1-cKO* mice (*n* = 6, Student’s *t* test) (**D**). Relative frequency distribution histograms show that the distribution curve corresponding to the *Ren1^c^-KO Itgb1-cKO* mice is displaced to the left of the control mice (**D**). (**E**) ISH for *Akr1b7* mRNA in kidneys from *Ren1^c^-KO Itgb1-c-KO* and *Ren1^c^-KO* control mice. The number of *Akr1b7* mRNA-positive cells in *Ren1^c^-KO Itgb1-cKO* mice is much smaller than in *Ren1^c^-KO* controls. Arrows indicate afferent arterioles. Scale bars: 50 μm. (**F**) GFP showed in the walls of hypertrophic afferent arterioles in the *Ren1^c^-KO* control (*Ren1^c^-KO^–/–^ Ren1^cCre/+^ Itgb1^fl/+^ R26mTmG*) but was absent in the arterioles of *Ren1^c^-KO Itgb1-cKO* kidneys (*Ren1^c^-KO^–/–^ Ren1^cCre/+^ Itgb1^fl/fl^ R26mTmG*) while persisting in some tubules. Scale bars: 50 μm. (**G**) PAS staining and Masson’s trichrome staining in kidneys from *Ren1^c^-KO* and *Ren1^c^-KO Itgb1-cKO* mice showed fibrosis in both groups. Scale bars: 50 μm. All data are reported as means ± standard deviation. Black triangles represent male samples and purple dots female samples. ****P < 0.0001. *Itgb1-cKO*, deletion of the *Itgb1* gene in cells of the renin lineage; JG, juxtaglomerular; PAS, periodic acid–Schiff; *Ren1^c^-KO*, *Ren1^c^* gene knockout.

Our scRNA-Seq results indicate that the integrin β_1_ gene (*Itgb1*) was highly upregulated in *Renin^null^* cells (Figure 6A and Supplemental Table 1). In fact, ISH showed that *Itgb1* mRNA expression not only was higher in the *Renin^null^* cells compared with WT renin cells but also extended along with the chronically recruited *Renin^null^* cells along the afferent arterioles (Figure 6A). To test the function of the integrin β_1_, we deleted *Itgb1* in the *Renin^null^* cells. We crossed *Ren1^c–/–^ Ren1^c^-Cre* mice with *Itgb1^fl^* mice and generated *Ren1^c–/–^* mice with conditional deletion of *Itgb1* in cells of the renin lineage (*Ren1^c^-KO Itgb1-cKO*; Figure 6B). The *Ren1^c^-KO Itgb1-cKO* mice were born at the expected Mendelian ratios and required subcutaneous injections of saline during the first week after birth to prevent dehydration and hypovolemia as are known to occur in *Ren1^c–/–^* mice (29). There was no difference in body weights and kidney weights, degree of renal insufficiency, or anemia between the *Ren1^c^-KO* mice (control group) and *Ren1^c^-KO Itgb1-cKO* mice (Supplemental Figure 6). The arteriolar hypertrophy was prevented in the *Ren1^c^-KO Itgb1-cKO* mice at 4 months of age (Figure 6, C and D). The wall thickness of the afferent arterioles in *Ren1^c^-KO Itgb1-cKO* mice was significantly smaller than in control *Ren1^c^-KO* mice (Figure 6D). This was also evident in the histogram of Figure 6D, demonstrating that the hypertrophy was prevented at all arteriolar sizes. To define whether the arteriolar hypertrophy was due to the presence/influence of the *Renin^null^* cells, we performed ISH for *Akr1b7,* a marker of renin cells that is expressed even when the *renin* gene is deleted. The expression of *Akr1b7* mRNA was substantially diminished in the afferent arterioles of the *Ren1^c^-KO Itgb1-cKO* mice (Figure 6E), indicating the successful ablation of most of the *Renin^null^* cells at 4 months of age. To confirm these findings, we crossed *Ren1^c^-KO Itgb1-cKO* mice with *mTmG* mice and traced the fate of GFP-positive (renin lineage) cells. In the control *Ren1^c^-KO* mice, GFP-positive cells were present in the hypertrophic afferent arterioles, as reported before (9). On the other hand, only a few GFP-positive cells were present in the afferent arterioles at each JG area in the kidneys of *Ren1^c^-KO Itgb1-cKO* mice (Figure 6F). These results indicate not only that integrin β_1_ was necessary for *Renin^null^* cells’ survival but also that the null cells were responsible for the arteriolar hypertrophy elicited by the lack of renin. Periodic acid–Schiff (PAS) staining and Masson’s trichrome staining also showed the lack of arteriolar hypertrophy in *Ren1^c^-KO Itgb1-cKO* mice (Figure 6G). Interestingly, the degree of interstitial fibrosis was similar to the one found in control *Ren1^c^-KO* mice, suggesting the possibility that more time may be required to prevent the interstitial disease or that additional pathogenic mechanisms are involved in the interstitial disease. However, the periglomerular fibrosis was milder in *Ren1^c^-KO Itgb1-cKO* mice than control *Ren1^c^-KO* mice (Figure 6C).

### Long-term inhibition of RAS induces arteriolar hypertrophy in mice.

**Figure 7:**
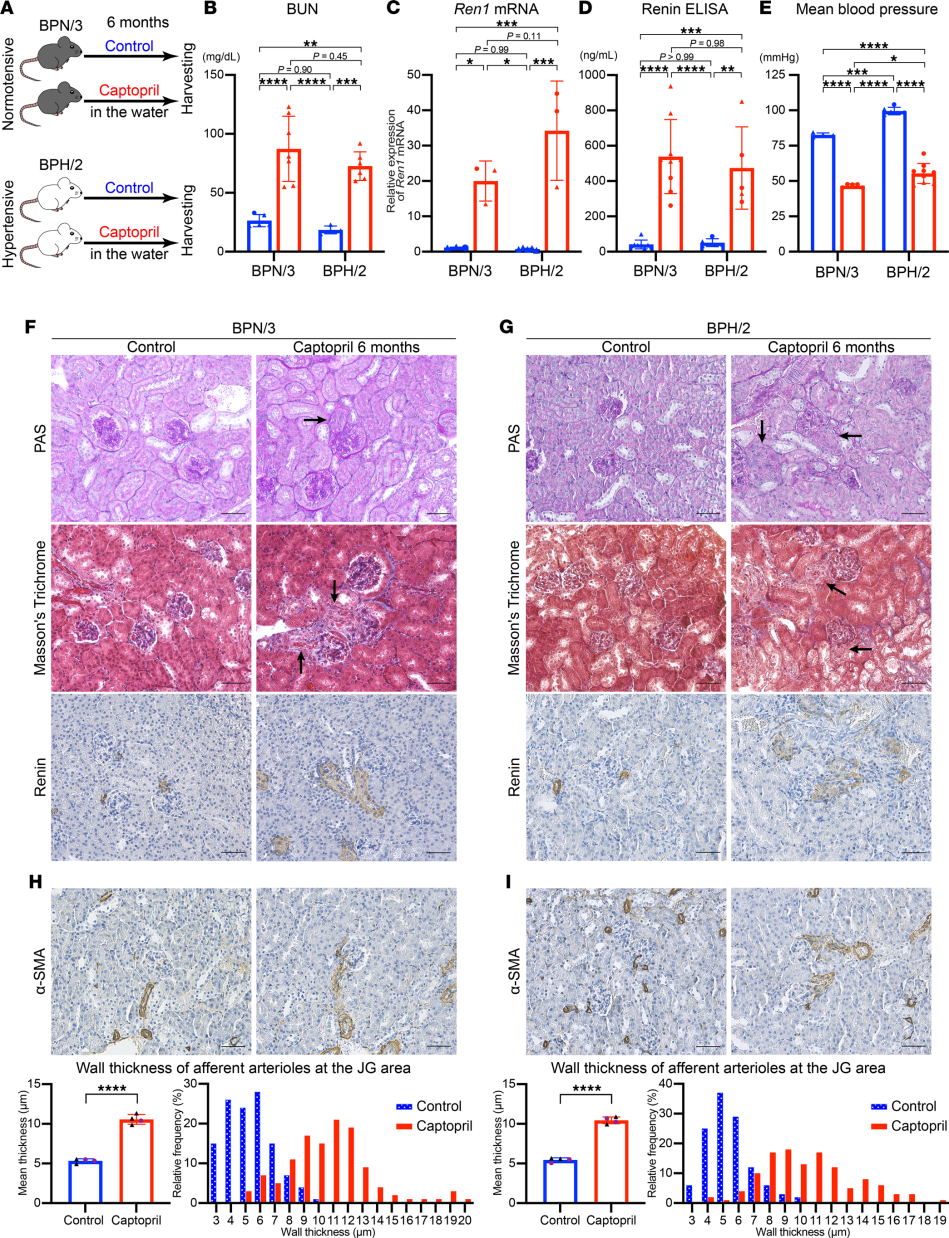
Long-term inhibition of RAS induces arteriolar hypertrophy in mice. (**A**) Schematic of the treatments of BPN/3 and BPH/2 mice. After 6 months of treatment with captopril in the drinking water, BUN (**B**, *n* ≥ 4), *Ren1* mRNA (**C**, *n* ≥ 3), and plasma renin (**D**, *n* ≥ 5) were significantly increased in both BPN/3 and BPH/2 mice (2-way ANOVA followed by Tukey’s multiple comparison test). (**E**) The mean blood pressure was significantly decreased by the captopril treatment in both BPN/3 and BPH/2 mice (*n* ≥ 4, 2-way ANOVA followed by Tukey’s multiple comparison test). Arteriolar hypertrophy induced by renin cells with long-term captopril treatment in BPN/3 mice (**F**) and BPH/2 mice (**G**) shown by PAS staining, Masson’s trichrome staining, and immunohistochemistry for renin. Arrows indicate hypertrophic afferent arterioles. Scale bars: 50 μm. Immunohistochemistry for α-SMA showed increased thickness of the walls of afferent arterioles in kidneys from BPN/3 mice (**H**) and BPH/2 mice (**I**). The mean wall thickness of afferent arterioles at the JG area was significantly larger in mice with long-term captopril treatment (*n* = 4, each, Student’s *t* test). Relative frequency distribution histograms show that the distribution curves corresponding to the mice with captopril treatment are displaced to the right of controls. Scale bars: 50 μm. All data are reported as means ± standard deviation. Triangles represent male samples, and dots female samples. *P < 0.05, **P < 0.01, ***P < 0.001, ****P < 0.0001. BUN, blood urea nitrogen; JG, juxtaglomerular; PAS, periodic acid–Schiff; RAS, renin-angiotensin system.

Deletion of the *renin* gene leads to strong activation of the *Renin^null^* cells, resulting in arteriolar hypertrophy. Given our results with gene deletion studies, we hypothesized that animals with long-term pharmacological inhibition of the RAS would also display chronic activation of renin cells and arteriolar hypertrophy. To test these hypotheses, we used genetically hypertensive mice (BPH/2) and their normotensive control mice (BPN/3). We treated both BPN/3 and BPH/2 mice with captopril in the drinking water for 6 months and compared them with mice not receiving captopril (Figure 7A and Supplemental Figure 7). All mice received a normal diet and water ad libitum. Both BPN/3 and BPH/2 mice showed elevated blood urea nitrogen after the treatment (Figure 7B). Long-term usage of captopril increased renin and its mRNA in both BPN/3 and BPH/2 mice (Figure 7, C and D). Blood pressure measurements under anesthesia showed a significantly decreased mean blood pressure in mice treated with captopril compared with untreated controls in both BPN/3 and BPH/2 mice (Figure 7E). The long-term usage of captopril induced marked afferent arteriolar hypertrophy in both BPN/3 (Figure 7F) and BPH/2 mice (Figure 7G). The wall thickness of the afferent arterioles in both BPN/3 and BPH/2 mice treated with captopril was significantly increased when compared with the untreated controls (BPN/3: control; 5.54 ± 0.27 μm vs. captopril; 10.57 ± 0.61 μm, *P* < 0.0001, BPH/2: control; 5.46 ± 0.26 μm vs. captopril; 10.44 ± 0.43 μm, *P* < 0.0001) (Figure 7, H and I). The hypertrophic arterioles were covered along their lengths with cells positive for renin (Figure 7, F and G), suggesting that renin cells were involved in the hypertrophy.

### In human patients, long-term inhibition of the RAS induces arteriolar hypertrophy.

**Figure 8:**
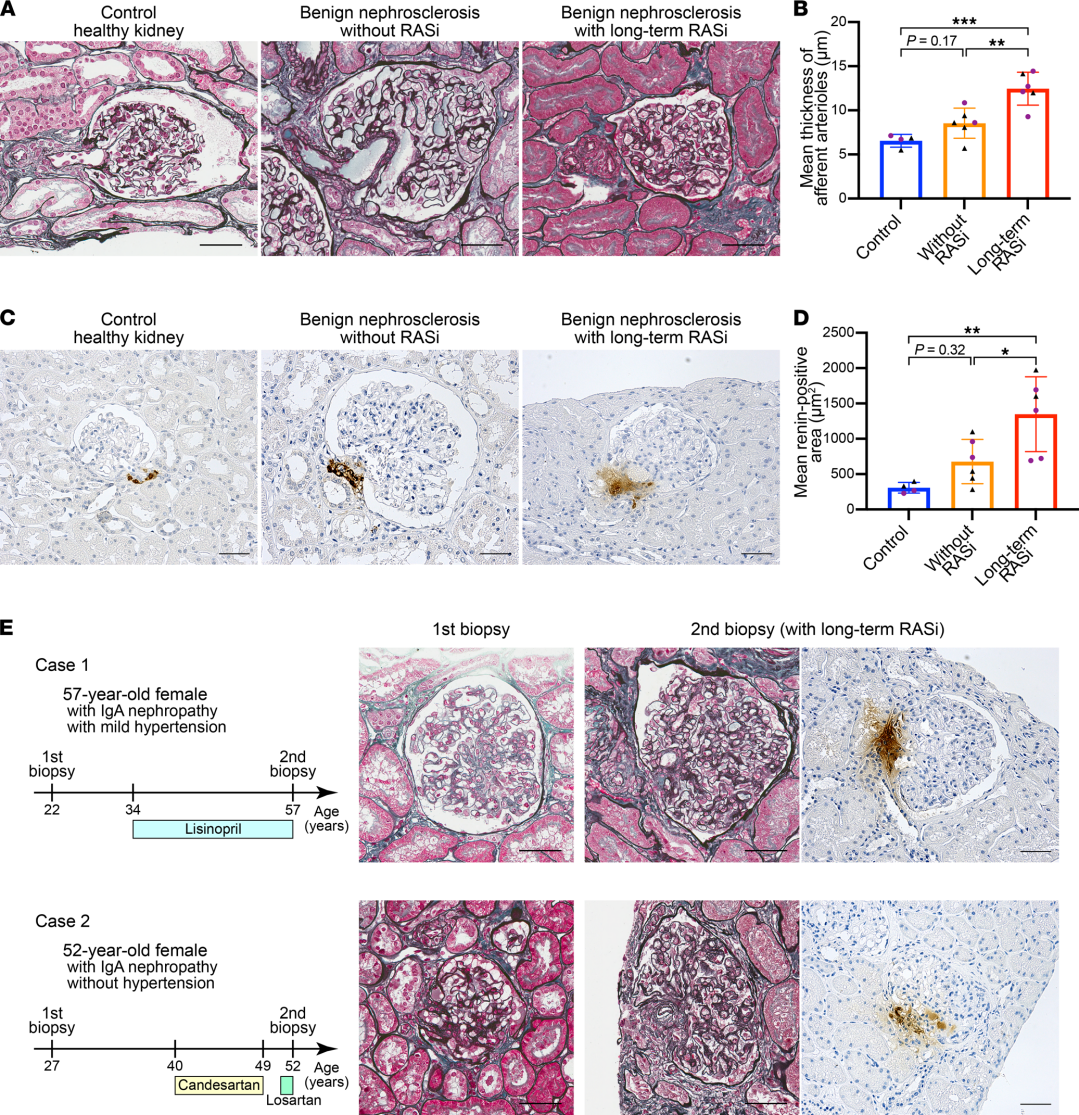
Long-term inhibition of RAS induces arteriolar hypertrophy in human patients. (**A**) Representative images of periodic acid–methenamine silver (PAM) with Masson’s trichrome staining of kidney sections from healthy control individuals, patients with benign nephrosclerosis without RASi, and patients with benign nephrosclerosis with long-term usage of RASi. Patients with long-term usage of RASi showed marked hypertrophy in afferent arterioles. (**B**) Mean thickness of afferent arterioles at the JG area. Patients with long-term usage of RASi (*n* = 6) showed significantly thicker renal arteriolar walls compared with controls (*n* = 4) and patients with nephrosclerosis without RASi (*n* = 6) (1-way ANOVA followed by Tukey’s multiple comparisons test). (**C**) Representative images of immunohistochemistry for renin in kidney sections. Patients with long-term usage of RASi showed extensive renin-positive regions in the JG area. (**D**) Mean renin-positive area at the JG area. Patients with long-term usage of RASi (*n* = 6) showed significantly larger areas compared with controls (*n* = 4) and patients with nephrosclerosis without RASi (*n* = 6) (1-way ANOVA followed by Tukey’s multiple comparisons test). (**E**) Patients with long-term RASi with mild or no hypertension. Patient 1 was a 57-year-old female with IgAN. She was diagnosed with IgAN by her first renal biopsy and administered lisinopril (ACEi) for 23 years before her second renal biopsy. Patient 2 was a 52-year-old female with IgAN. She was diagnosed with IgAN by her first renal biopsy and administered candesartan (ARB) for 9 years. After 2 years of self-interruption, she received losartan (ARB) for 1 year before her second renal biopsy. Both patients had no abnormalities in afferent arterioles at their first biopsies and showed marked afferent arteriolar hypertrophy at the second renal biopsies. Scale bars: 50 μm. All data are reported as means ± standard deviation. *P < 0.05, **P < 0.01, ***P < 0.001. ACEi, angiotensin-converting enzyme inhibitor; ARB, angiotensin II receptor blocker; JG, juxtaglomerular; IgAN, IgA nephropathy; RAS, renin-angiotensin system; RASi, renin-angiotensin system inhibitor.

We then investigated the influence on the activation of renin cells by the inhibition of RAS in humans. We studied previously performed renal biopsy samples from (a) 6 patients with benign nephrosclerosis who underwent long-term inhibition of the RAS for more than 5 years, (b) 6 age-matched patients with benign nephrosclerosis who had never received RASi, and (c) 4 healthy controls (Supplemental Table 7). There was no difference in renal function, urinary protein, and blood pressure between the benign nephrosclerosis patients with long-term usage of RASi and without RASi in this study (Supplemental Figure 8). Patients with long-term usage of RASi showed significantly thicker kidney arteriolar walls compared with healthy controls and patients with nephrosclerosis without RAS inhibition (control; 6.55 ± 0.73 μm, without RASi; 8.54 ± 1.71 μm, vs. long-term RASi; 12.46 ± 1.86 μm, *P* < 0.001) (Figure 8, A and B). To test whether renin cells were involved in arteriolar hypertrophy in humans, we immunostained for renin in kidney sections from the same patients mentioned above. We found that the renin-positive area was significantly increased by long-term usage of RASi (control; 307.3 ± 74.7 μm^2^, without RAS; 677.8 ± 313.4 μm^2^, vs. long-term RAS; 1347 ± 529.9 μm^2^, *P* = 0.003; Figure 8, C and D).

Also, we had access to samples from 2 patients with IgA nephropathy (IgAN) who underwent renal biopsies before receiving RASi. The first renal biopsies showed no abnormalities in the afferent arterioles. Thereafter, these patients received an angiotensin-converting enzyme inhibitor (ACEi) or an angiotensin II receptor blocker (ARB) as a treatment for IgAN; neither patient experienced severe hypertension. They underwent their second renal biopsies after more than 10 years of RAS inhibition because of declines in renal function and proteinuria. Regardless of the long disease course of IgAN, the glomerular damage remained mild, and the glomeruli were maintained. However, we found that they developed severe afferent arteriolar hypertrophy (Figure 8E) in parallel with an expansion of the renin-positive areas over the visible portion of the arterioles and JG apparatus.

Thus, inhibition of the RAS in humans leads to stimulation of renin cells and concentric hypertrophy of the preglomerular arterioles.

## Discussion

The present studies show that deletion of renin or inhibition of the RAS in mice and humans leads to concentric thickening of the intrarenal arteries and arterioles. This severe disease is caused by the multiclonal expansion and transformation of renin cells from a classical endocrine phenotype to an invasive matrix-secretory phenotype: the cells surround and invade the vessel walls and stimulate the accumulation of adjacent SMCs, resulting in blood flow obstruction, localized ischemia, and fibrosis. Ablation of the renin cells via long-term conditional deletion of β_1_ integrin prevents the arteriolar disease, indicating that renin cells are fully responsible for the arteriolar hypertrophy. In those mice at 4 months of age, the number of renin lineage cells was markedly decreased, consistent with the known role of *Itgb1* in the prevention of anoikis and apoptosis in renin lineage cells (30). Similar results are obtained when renin cells are ablated in mice using diphtheria toxin genetically engineered to be expressed under the control of the *renin* gene (31). Thus, renin cells are a sine qua non for the arterial pathology to occur. It would be interesting to examine whether a controlled manipulation of integrin β_1_ expression could prevent this and other vascular diseases.

As indicated above, our lineage tracing experiments using the confetti mice reveal that *Renin^null^* cells are of multiclonal origin. Thus, the expansion of these cells is not due to the selective clonal advantage of one cell population over another in the pathogenesis of vascular disease. The process seems to be stochastic, with each individual cell having equal chances to participate in the cell transformation to a disease phenotype. Nevertheless, this issue needs to be studied in more detail. Further, the increase in cell number along the renal arterioles is not due to cell proliferation but instead to a phenotypic switch in cell identity, as we have previously suggested (9). This is in agreement with a recent study showing that exposure of mice to a low-salt diet and captopril, which increases the number of renin cells along the arterioles, does not result in renin cell proliferation. Instead, the cells upregulate the expression of antiproliferative genes, suggesting the plastic transformation of SMCs to the renin phenotype (22).

To understand how these cells contribute to the arterial pathology, we examined the transcriptome of individual *Renin^null^* cells. In addition to their remarkable morphological transformation, the transcriptome of *Renin^null^* cells was strikingly different from the transcriptome of WT cells. Their TF repertoire suggested the cells sustained cellular stress as indicated by the expression of *Ddit3*, known to regulate ER stress–induced apoptosis (20). Also, the cells expressed *Klf2*, which has been reported to be involved in the inhibition of cell proliferation (21), indicating that these cells are not proliferative and they increase their numbers by phenotypic switching and cell transformation. In addition to their unique circuitry of TFs, the null cells synthesize a large variety of secreted proteins. In fact, *Renin^null^* cells express over 58 genes encoding secreted proteins versus only 6 in WT renin cells. Our bioinformatic analysis indicated that these secreted proteins are likely involved in the restructuring of the composition of the extracellular matrix, as it occurs in amyloid fibril formation and wound healing. Thus, *Renin^null^* cells not only change their phenotype but also alter the composition and structure of their own extracellular environment. In doing so, they also affect the behavior and fate of SMCs, which accumulate concentrically among excessive layers of basement membrane–like material, leading to the inexorable obstruction of blood flow. It would be interesting to examine whether those secreted proteins (*Bgn,* *Cpe,* *Emillin1,* *Fstl1,* *Nid1,* *Mgp,* *Sparc,* and *Spp1*) validated by our ISH are the result of the arterial disease or play a direct causative role. Of interest, *Spp1* is not only present in the diseased arterioles but also elevated in the circulation of *Ren1^c^-KO* mice. Further studies will be necessary to determine whether osteopontin, together with any of the secretory proteins we identified, can be used to predict and mark the progression of arterial disease.

The findings of this study are not only fundamental from a basic science standpoint but also relevant from a medical perspective. We found that long-term pharmacological inhibition of the RAS induces arteriolar hypertrophy not only in mice but also in humans. Drugs affecting the RAS have been used for the treatment of patients with a variety of diseases, including congestive heart failure, myocardial infarction, hypertension, and diabetic nephropathy, and for reducing the risk of developing major cardiovascular events (32). On the other hand, dual blockade of the RAS with ARBs and ACEi may result in a reduction of proteinuria but worsen major renal outcomes (33), and a direct renin inhibitor in combination with an ACEi or ARB was reported to be harmful (34). Those studies implied some untoward effects caused by the inhibition of the RAS. We showed that both normotensive and hypertensive mice with long-term RASi developed a marked increase in numbers of renin-positive cells at the JG area. We previously reported that dual blockade of RAS using aliskiren and valsartan (ARB) to congenic mRen2.Lewis hypertensive rats showed improvements in the surrogate endpoints of blood pressure, ventricular mass, and proteinuria but also showed tubular cell damage, interstitial and periglomerular fibrosis, and afferent arteriolar hypertrophy (11). In this study, we also found that the long-term inhibition of the RAS induced arteriolar hypertrophy in humans. Some case reports showed hypertrophy of the JG area in patients with hypertension treated with RASi (35). In a recent study of the kidneys from hypertensive patients treated with antihypertensive agents, arteriolar hypertrophy was observed in patients with RASi and not detected in patients without RASi (12). Here, we report the presence of arteriolar hypertrophy in patients without hypertension but with long-term inhibition of the RAS. In these patients with IgAN who were treated with RASi without hypertension, renal function was maintained for a prolonged period of time. Therefore, RASi may have been of benefit for the treatment of IgAN. However, treatment with RASi was associated with afferent arteriolar hypertrophy. The number of samples with long-term RASi in this cross-sectional study was not enough to determine the effects on renal function. Therefore, further studies — using sensitive techniques to measure renal function — are needed to examine the long-term effect of RAS inhibition on renal prognosis.

In summary, we found that renin cells are responsible for arteriolar hypertrophy when they are chronically stimulated by either experimental or spontaneous mutations of the RAS genes or the use of RASi in mammals, including humans. Whereas we do not advocate to stop using RASi — which are effective to treat hypertension and prevent cardiovascular disease and/or mortality — given our results and those of other studies cited here, it would be important to conduct prospective randomized, controlled studies with histological evaluation to determine the extent of morphological and functional renal damage caused by the widespread use of RASi.

## Methods

### Animals.

All animals were maintained in a room with controlled temperature and humidity under a 12-hour light/12-hour dark cycle. *Ren1^c^-KO* mice were generated as previously reported (29). To generate *Ren1^c–/–^ Ren1^c^-Cre R26R-Confetti* mice, we crossed *Ren1^c–/–^* mice with the *Ren1c-Cre* mice (36) and the *R26R-Confetti* mice (Jackson Laboratory: 013731). scRNA-Seq of WT renin cells and *Renin^null^* cells was performed in the Fluidigm C1 system, using the *Ren1^c^-YFP* mice (15). We used 4 *Ren1c^+/+^* *Ren1c-YFP* mice and 12 *Ren1c^–/–^ Ren1c-YFP* mice at adult ages. To perform scRNA-Seq of *Renin^null^* cells and other vascular cells, we generated *Ren1^c^-KO SMMHC-CreER^T2^ tdTomato Ren1^c^-YFP* mice. Two male mice were injected 0.125 g/body weight of tamoxifen at 5 weeks of age and used at 8 weeks of age. *SMMHC-CreER^T2^* mice (Jackson Laboratory: 019079) were provided by Brant E. Isakson (Robert M. Berne Cardiovascular Research Center, University of Virginia School of Medicine, Charlottesville, Virginia, USA). *tdTomato* mice were obtained from Jackson Laboratory (007909). To generate *Ren1^c^-KO Itgb1c-KO* mice, we used the *Ren1^c^-Cre* mice and the *Itgb1*^fl^ mice (Jackson Laboratory: 004605). We tested *Ren1^c^-KO Itgb1-cKO* mice at 4 months of age. BPN/3 and BPH/2 mice were obtained from Jackson Laboratory (003004 and 003005, respectively). Genotyping of the mice was performed by Transnetyx. Both male and female mice were used in the experiments. All mice with *Ren1^c^* KO allele received subcutaneous injections of saline on 5 consecutive days after birth to ensure their survival.

### Histological analysis of mouse kidneys.

Mice were anesthetized with tribromoethanol (300 mg/kg). Kidneys were removed; fixed in 4% paraformaldehyde (PFA), 10% formalin, or Bouin’s solution overnight; and embedded in paraffin. PAS staining and Masson’s trichrome staining were performed in 5 μm paraffinized sections of kidneys fixed with Bouin’s solution.

For frozen sections of Confetti, YFP, and tdTomato mice, kidneys were fixed in 4% PFA for 1 hour at 4°C. After washing, the kidneys were incubated in 30% sucrose overnight at 4°C and frozen in O.C.T. (Thermo Fisher Scientific). The frozen blocks were sectioned at 20 μm thickness and washed in PBS. Staining for nuclei was performed with Hoechst 33342 (Thermo Fisher Scientific). Sections were covered with cover glasses with PBS.

### Microscopy.

Kidney sections were visualized using a Zeiss Imager M2 microscope equipped with an ApoTome-2 fitted with the AxioCam 305 color and AxioCam 506 mono camera (Zeiss).

### Immunohistochemistry.

Sections from Bouin’s-fixed, paraffin-embedded kidneys were deparaffinized, rehydrated, and treated with 0.3% hydrogen peroxide in methanol. After blocking with 3% BSA and 2% goat serum or horse serum, sections were incubated with the anti-renin antibody (rabbit polyclonal anti-mouse antibody; diluted at 1:500) or the anti–α-SMA antibody (catalog A2547 [MilliporeSigma]; diluted at 1:10,000) at 4°C overnight. After washing, sections were incubated with biotinylated secondary antibody, goat anti–rabbit IgG (catalog BA-1000 [Vector Laboratories]; diluted at 1:200) or horse anti–mouse IgG (BA-2000 [Vector Laboratories]; diluted at 1:200) for renin or α-SMA, respectively, at room temperature for 30 minutes. Staining was amplified using the Vectastain ABC kit (Vector Laboratories) and developed with 3,3′-diaminobenzidine (MilliporeSigma). The sections were counterstained with hematoxylin (MilliporeSigma), dehydrated, and mounted with Cytoseal XYL (Thermo Fisher Scientific). For the staining of renin in human sections, the recombinant anti-renin antibody (catalog ab212197 [Abcam]; diluted at 1:2000) was used as the primary antibody.

The wall thickness of the afferent arterioles was measured in the kidney sections stained for α-SMA using Zen software (Zeiss). For each sample, we selected 30 afferent arterioles that were directly connected to the glomeruli and measured the outer and inner diameters of the arterioles. Wall thickness was then calculated as the difference between the 2 diameters divided by 2.

### EM.

For EM, kidneys were fixed in 3% glutaraldehyde in 0.1 M phosphate buffer, pH 7.3. The tissue was washed in the same buffer for 15 minutes, 3 times, prior to secondary fixation with 1% osmium tetroxide in 0.1 M phosphate buffer for 1 hour at room temperature. The tissues were dehydrated using a graded ethanol series, transitioned through toluene, infiltrated and embedded with Poly/Bed 812 epoxy resin, and polymerized overnight at 60°C. Areas of interest for EM were selected from thick sections (1 μm) stained with toluidine blue. Thin sections (100 nm) were cut with a diamond knife (Diatome) and placed on 200 mesh nickel grids for staining with uranyl acetate and lead citrate.

### Synthesis of probes for ISH.

To develop the probes for ISH, DNA fragments were synthesized by PCR using cDNA from WT C57BL/6 mouse kidneys and primers containing a 3′ T3 promoter and a 5′ T7 promoter sequence. Primers are listed in Supplemental Table 8. After purification and confirmation of DNA sequences, digoxigenin-labeled (DIG-labeled) RNA sense and antisense probes were generated by in vitro transcription using DIG RNA Labeling Mix and T3 or T7 Polymerase (MilliporeSigma) (37).

### ISH.

ISH was performed in 4% PFA-fixed, paraffin-embedded kidney tissues. The tissues were sectioned at 7 μm thickness, deparaffinized in xylene, rehydrated with sequential incubations in ethanol (100%, 95%, and 70%), washed with PBS, and postfixed with 4% PFA at room temperature for 30 minutes, followed by acetylation (0.375% acetic anhydride) for 10 minutes and permeabilization with proteinase K (10 μg/mL) for 30 minutes at 37°C. After preincubation with hybridization buffer (500 ng/mL in hybridization buffer of 50% formamide, 5× SSC, 50 μg/mL yeast transfer RNA, 1% SDS, 50 μg/mL heparin) for 1 hour at 37°C, sections were incubated with the DIG-labeled sense or antisense riboprobes at 55°C overnight. The sections were washed 3 times with 0.2× SSC at 65°C for 30 minutes, blocked with 10% heat-inactivated sheep serum for 1 hour, and then incubated with anti–digoxigenin-alkaline phosphatase antibody (1:4000, catalog 11093274910 [MilliporeSigma]) overnight at 4°C. After washing, sections were treated with solution (100 mmol/L NaCl, 100 mmol/L Tris pH 9.5, 50 mmol/L MgCl_2_, 0.1% Tween-20, 2 mmol/L levamisole) for 10 minutes. Sections were incubated with BM Purple (MilliporeSigma) for 3 hours to 7 days with careful observation of the signals. Sections were fixed by 0.2% glutaraldehyde + 4% PFA and mounted with Glycergel Mounting Medium (Agilent Technologies). We confirmed the specificity of ISH by comparing antisense probes generated with T7 polymerase and sense probes generated with T3 polymerase. The ISH signals were observed with antisense probes, and there was no signal with sense probes for all genes we targeted. To compare the intensity of the signals, we placed the kidney sections of both the right and left kidneys from the same mice on the same slides and treated the sections equally during the whole procedure (37).

### Isolation of single cells.

Cells were isolated from the kidney cortex using FACS (9). The kidneys were excised and decapsulated. Then, the kidney cortices were dissected, minced with a razor blade, and transferred into a 15 mL tube with 5 mL of enzymatic solution (0.3% collagenase A [MilliporeSigma], 0.25% trypsin [MilliporeSigma], and 0.0021% DNase I [MilliporeSigma]). The tubes were placed flat inside a shaking incubator (80 rpm) for 15 minutes at 37°C. The solution was pipetted up/down 10 times with a sterile transfer pipette and allowed to settle for 2 minutes, and the supernatant was collected in a fresh tube on ice. The enzymatic solution was added to the 15 mL tube containing the remaining undigested cortices, and the digestion procedure was repeated 3 times. The supernatants were pooled and centrifuged at 800*g* for 4 minutes at 4°C using a Sorvall RT7 refrigerated centrifuge (Sorvall). The cell pellet was resuspended in fresh buffer 1 (130 mM NaCl, 5 mM KCl, 2 mM CaCl_2_, 10 mM glucose, 20 mM sucrose, 10 mM HEPES, pH 7.4), and the suspension was poured through a sterile 100 μm nylon cell strainer (Corning Inc.) and washed with buffer 1. The flow-through was poured through a sterile 40 μm nylon cell strainer (Corning Inc.) and washed with buffer 1. The flow-through was centrifuged at 1100*g* for 4 minutes at 4°C. The cell pellet was resuspended in 1.5 mL of resuspension buffer: PBS, 1% FBS, 1 mM EDTA, DNase I (MilliporeSigma). The dead cells were labeled with DAPI (MilliporeSigma). Cells were analyzed and sorted using an Influx Cell Sorter (Becton Dickinson) at the Flow Cytometry Core Facility of the University of Virginia. Cells were collected in DMEM with 10% FBS and used for single-cell capture and downstream applications.

### Single-cell capture and mRNA sequencing by the Fluidigm C1 System.

Sorted YFP-positive cells were loaded onto the C1 Fluidigm System (Fluidigm) according to the manufacturer’s instructions. Briefly, a medium-sized integrated fluidic circuit (IFC 10–15 μm) was primed with Fluidigm reagents. Cells were suspended at a concentration of approximately 400 cells/μL and mixed with Fluidigm suspension reagent at a ratio of 3:2, to a total volume of 20 μL. Six microliters of the cell/suspension mix was then loaded into the IFC. At the end of the capture, wells containing single viable cells were identified by visual inspection with a 10× bright-field microscope (DM5500 B, Leica). Lysis, RT, and PCR solutions from the SMART-Seq v4 Ultra Low Input RNA Kit for the Fluidigm C1 System (Takara Bio USA, Inc.) and Fluidigm reagents were loaded onto the IFC as described by the manufacturer. We conducted single-cell profiling of 51 WT renin cells and 96 *Renin^null^* cells. The cDNA was diluted with Fluidigm dilution buffer and stored at –20°C. These steps were repeated for every independent capture of cells. In total, we conducted 4 captures for *Ren1^c–/–^* *Ren1^c^-YFP* cells and 1 capture for *Ren1^c+/+^* *Ren1^c^-YFP* cells. Samples underwent next-generation sequencing library preparation using the Nextera XT DNA Library Preparation Kit (Illumina). Briefly, cDNA samples were diluted, tagmented, and indexed with unique barcodes for downstream analyses. After the addition of indices and amplification, samples were multiplexed and cleaned using the Agencourt AMPure XP Kit (Beckman Coulter). Libraries were sequenced on an Illumina HiSeq 2500/4000 platform (150 bp paired-end reads).

Sequencing data were initially quality checked using FastQC with the FASTQ file reads. Prior to alignment, we removed low-quality reads and adapter sequences using Trimmomatic (38). We aligned FASTQ reads to the GRCm38/ENSEMBL mouse genome using Salmon (39), and transcript-level estimates of expression were scaled up to gene-level estimates using the Tximport R package (40), with the lengthScaledTPM argument for abundance estimation.

### Single-cell capture and mRNA sequencing by 10x Genomics Chromium System.

The tdTomato-YFP double-positive cells, tdTomato-positive cells, and YFP-positive cells were isolated by FACS. Single cells were captured with the Chromium System (10x Genomics) following the manufacturer’s recommendation. Briefly, sorted cells were washed with PBS containing 0.04% BSA and loaded into a Chromium Next GEM Chip G with reagents of Chromium Next GEM Single Cell 3′ Reagent Kits v3.1 (10X Genomics). The Cell-Gel Beads in Emulsion were generated and incubated to generate the barcoded cDNA. cDNA was cleaned with Dynabeads (10x Genomics) and amplified with 12 cycles of PCR. cDNA was then enzymatically fragmented, end-repaired, poly-A–tailed, adapter-ligated, and amplified by PCR. The constructed libraries were sequenced on an Illumina HiSeq 2500/4000 platform (150 bp paired-end reads).

The FASTQ file from the sequencing was processed by Cell Ranger v4.0.0 (10x Genomics). For alignment, we made a custom reference package including YFP and tdTomato with mouse genome (mm10). Cellranger count was used for read alignment and gene expression quantification.

### scRNA-Seq data analysis.

R v.4.0.3 was used for graphical and statistical analysis. The R package Seurat v4.0.0 (41) was used for analyzing the data.

For scRNA-Seq analysis of WT renin cells and *Renin^null^* cells using the C1 System, we normalized the counts with removing the variation in mitochondrial mapping percentage using the SCTransform function (42). GO (43) of gene sets was obtained using Metascape (44). Pathway enrichment analysis (GO biological processes) was performed with the DEGs. The list of putative TFs for *Mus musculus* was obtained from the Animal Transcription Factor Database 3.0 (45). The PPI enrichment analysis was performed using the Molecular Complex Detection algorithm (46) in Metascape and visualized by Cytoscape (47). The list of the potential secreted protein genes was extracted from the reviewed secreted protein gene list in the Uniprot protein database (48).

For scRNA-Seq analysis of *Ren1^c^-KO SMMHC-CreER^T2^ tdTomato Ren1^c^-YFP* mice using the Chromium System, we utilized the filtered gene-barcode matrix generated by Cell Ranger. Cells with mitochondrial gene percentages > 25%, unique gene counts < 300, and unique molecular identifiers < 10,000 were discarded. The resulting gene expression matrix was normalized with a scale factor, 10,000 molecules per cell, and log transformed. Clustering was performed using highly variable genes using Seurat’s default settings. The UMAP was generated using 7 principal components from principal component analysis with a perplexity of 7 and a resolution of 0.2. The DEGs between clusters were determined using the FindMarkers function in Seurat. The trajectory analysis was performed using Monocle 3 (49) with a conversion function in the SeuratWrappers package. The analysis of the cAMP pathway was performed using the genes extracted from the Kyoto Encyclopedia of Genes and Genomes database (50).

### Long-term captopril treatment.

We treated the mice at adult ages with captopril added to the drinking water (0.5 g/L) for 6 months. During the treatment, a normal diet was given to mice, and the mice could freely access the diet and water bottles.

### Blood chemistry.

Animals were anesthetized by tribromoethanol (300 mg/kg). Blood was collected by cardiac puncture and placed into tubes containing EDTA or heparinized plasma separator tubes (BD Microtainer, Becton Dickinson). Tests of the basic metabolic panel were performed by the University of Virginia Hospital clinical laboratory (9).

### ELISA for renin and osteopontin in plasma samples.

Plasma specimens were obtained from blood after centrifugation at 1000*g* at 4°C for 15 minutes. Renin concentration was determined using ELISA (RayBiotech; ref. 51). ELISA for osteopontin was performed with Mouse/Rat Osteopontin (OPN) Quantikine ELISA Kit (R&D Systems).

### Blood pressure measurement.

Mice were anesthetized with 1.5% isoflurane and kept at 37.5°C. Polyethylene catheters (PE10, Becton Dickinson; internal diameter, 0.28 mm) prefilled with heparinized saline were inserted into right carotid arteries. Arterial pressure was continuously recorded simultaneously by an RX104A transducer with AcqKnowledge software (BIOPAC Systems Inc.). Mean arterial pressure was measured for 10 minutes (17).

### Immunofluorescence staining.

Sections at 5 μm from kidneys fixed with 10% formalin were deparaffinized, rehydrated, and microwave-treated with 10 mmol/L sodium citrate buffer. After blocking with 3% BSA, 5% donkey serum, 0.04% cold fish skin gelatin, and 0.05% Triton X-100 in PBS, sections were incubated with the primary antibodies at room temperature for 90 minutes. Anti–integrin β_1_ antibody (ab179471 [Abcam]; diluted at 1:1000) and anti–α-SMA antibody (A2547 [MilliporeSigma]; diluted at 1:5000) were used. Sections were washed, blocked again, and incubated with Alexa Fluor 568–conjugated donkey anti-rabbit antibody (A10042 [Thermo Fisher Scientific]; diluted at 1:500) and Alexa Fluor 488–conjugated donkey anti-mouse antibody (A21202 [Thermo Fisher Scientific]; diluted at 1:500). After washes, nuclei were stained with Hoechst 33342 (Thermo Fisher Scientific). Sections were mounted in ProLong Gold Antifade Mountant (Thermo Fisher Scientific).

### RNA extraction and quantitative reverse transcription PCR.

Renal cortices were removed and placed in RNAlater Stabilization Solution (Thermo Fisher Scientific) overnight at 4°C and then stored at −20°C. RNA was extracted from kidneys using TRIzol reagent (Thermo Fisher Scientific) and RNeasy Mini Kit (Qiagen). Reverse transcription was performed using oligo(dT) primer and M-MLV Reverse Transcriptase (Promega) at 42°C for 1 hour according to the manufacturer’s instructions. Quantitative PCR was performed with SYBR Green I (Thermo Fisher Scientific) in a CFX Connect system (Bio-Rad Laboratories). PCR was performed with the following primers; *Ren1*, forward: 5′-ACAGTATCCCAACAGGAGAGACAAG-3′, reverse: 5′-GCACCCAGGACCCAGACA-3′; *Rps14*, forward: 5′-CAGGACCAAGACCCCTGGA-3′, reverse: 5′-ATCTTCATCCCAGAGCGAGC-3′. The mRNA expression of *Ren1* was normalized to *Rps14* expression, and the changes in expression were determined by the ΔΔCt method and were reported as relative expression compared with control mice (17).

### Human samples.

The renal biopsy slides stained with periodic acid–methenamine silver (PAM) with Masson’s trichrome staining were scanned by Aperio CS2 scanner (Leica Biosystems). Image capture and measurement of the thickness of afferent arterioles were performed with Aperio ImageScope software (Leica Biosystems). Renin-positive areas were measured in renin-immunostained sections by ImageJ software (NIH).

### Data availability.

scRNA-Seq data sets generated in this study can be accessed at the Gene Expression Omnibus public repository using the accession numbers GSE180873 and GSE180893.

### Statistics.

Statistical analysis was performed using GraphPad Prism version 9.0.0 (GraphPad Software). The data were analyzed for normal distribution using the Shapiro-Wilk test. Data were considered normally distributed if the *P* value was not less than 0.05. Normally distributed data are shown as means ± standard deviation. Student’s 2-tailed *t* test, 1-way ANOVA with Tukey’s multiple comparison test, or 2-way ANOVA with Tukey’s multiple comparison test or Sidak’s multiple comparison test were used. *P < 0.05, **P < 0.01, ***P < 0.001, and ****P < 0.0001 were considered significant. Sample sizes were determined based on the numbers required to achieve statistical significance.

### Study approval.

All animals were handled in accordance with the NIH *Guide for the Care and use of Laboratory Animals* (National Academies Press, 2011), and the study was approved by the Institutional Animal Care and Use Committee of the University of Virginia (protocol no. 2433).

Human kidney samples were obtained from biopsy samples previously performed and stored at Niigata University. This study was approved by the Institutional Review Board of Niigata University Graduate School of Medical and Dental Sciences, Niigata, Japan (approval no. G2019-0015), and carried out according to the principles of the Declaration of Helsinki.

## Author contributions

HW, AGM, EAB, XL, and SM performed experiments; HW, AGM, and EAB analyzed the data; SG and IN organized the renal biopsy samples and processed the tissue specimens; LJA performed EM and interpreted the EM findings; HW and EAB drafted the initial version of the manuscript; AGM, MLSSL, and RAG read, reviewed, redrafted, and edited the manuscript; MLSSL and RAG designed the study and supervised the project; and all authors approved the final version of the manuscript.

## Supplementary Material

Supplemental data

Supplemental tables 1-8

## Version 1 11/11/2021

In-Press Preview

## Version 2 12/22/2021

Electronic publication
