# Supplementary material for: Inhibition of the renin-angiotensin system causes concentric hypertrophy of renal arterioles in mice and humans
Source: JCI Insight. 2021 Dec 22;6(24):e154337. doi: 10.1172/jci.insight.154337 (PMC8783690; doi:10.1172/jci.insight.154337)
Supplement: Supplemental data [file jciinsight-6-154337-s106.pdf]

Supplemental Figure 1

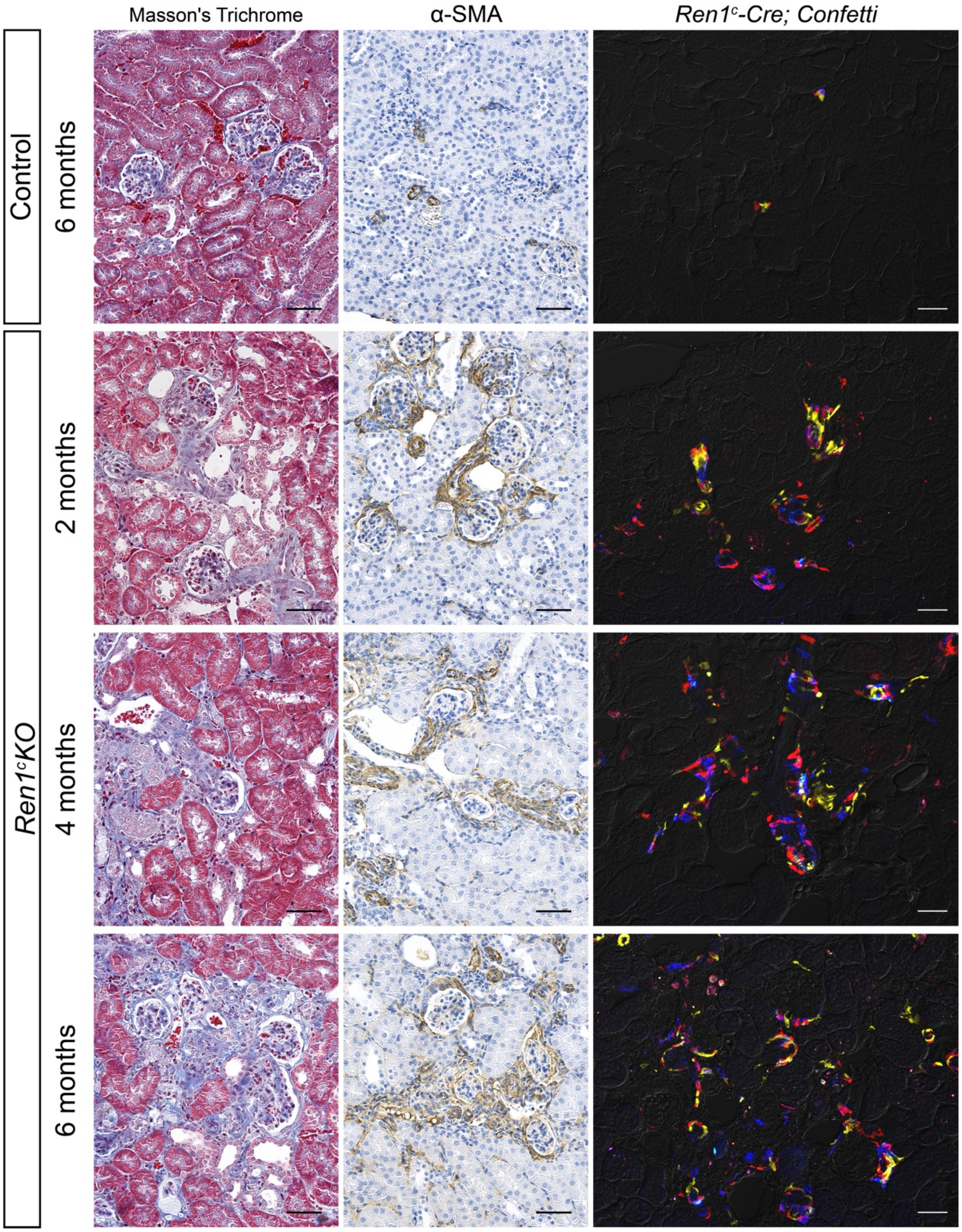

Supplemental Figure 1. Renin lineage cells contribute to arteriolar hypertrophy in a polyclonal way. Kidney tissues from *Ren1^{c}KO;Ren1^c-Cre;Confetti* and control mice. Masson's Trichrome staining, immunohistochemistry for α-SMA showed the progression of the renal disease with aging. Frozen sections showed that the policlonality was maintained during the disease progression. Scale bars, 50 μm. α-SMA; alpha-smooth muscle actin, *Ren1^{c}KO*; *Ren1^c* gene knockout.

Supplemental Figure 2

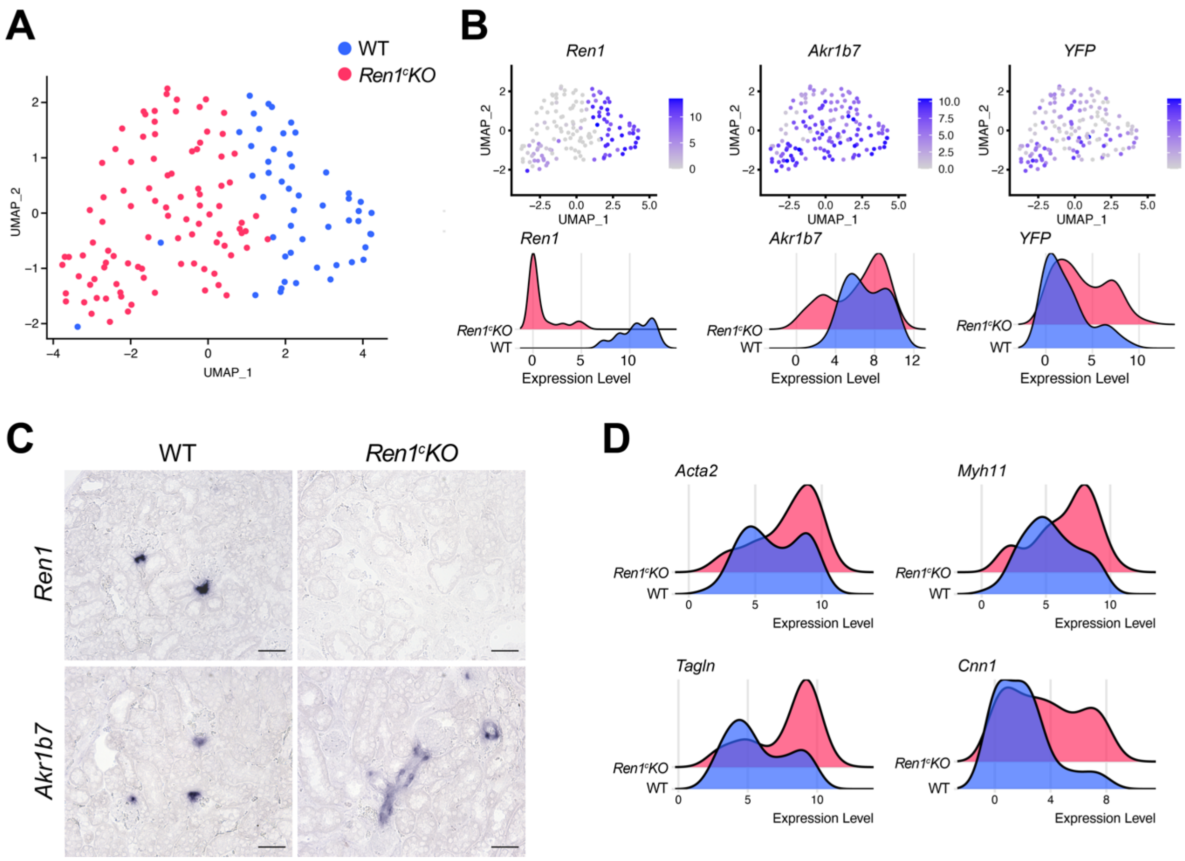

Supplemental Figure 2. Transcriptome of *Renin*^{null} cells with the Fluidigm C1 system. (**A**) The UMAP with all the cells after normalization. (**B**) Feature plots and ridge plots for *Ren1*, *Akr1b7*, and *YFP*. (**C**) ISH for *Ren1* and *Akr1b7* mRNA. *Akr1b7* positive cells were increased in kidneys from *Ren1^{c}KO* mice. Scale bars, 50 μm. (**D**) Ridge plots of smooth muscle genes (*Acta2*, *Mhy11*, *Tagln*, and *Cnn1*). ISH; *in situ* hybridization, *Ren1^{c}KO*; *Ren1^c* gene knockout, UMAP; Uniform Manifold Approximation and Projection, WT; wild-type.

Supplemental Figure 3

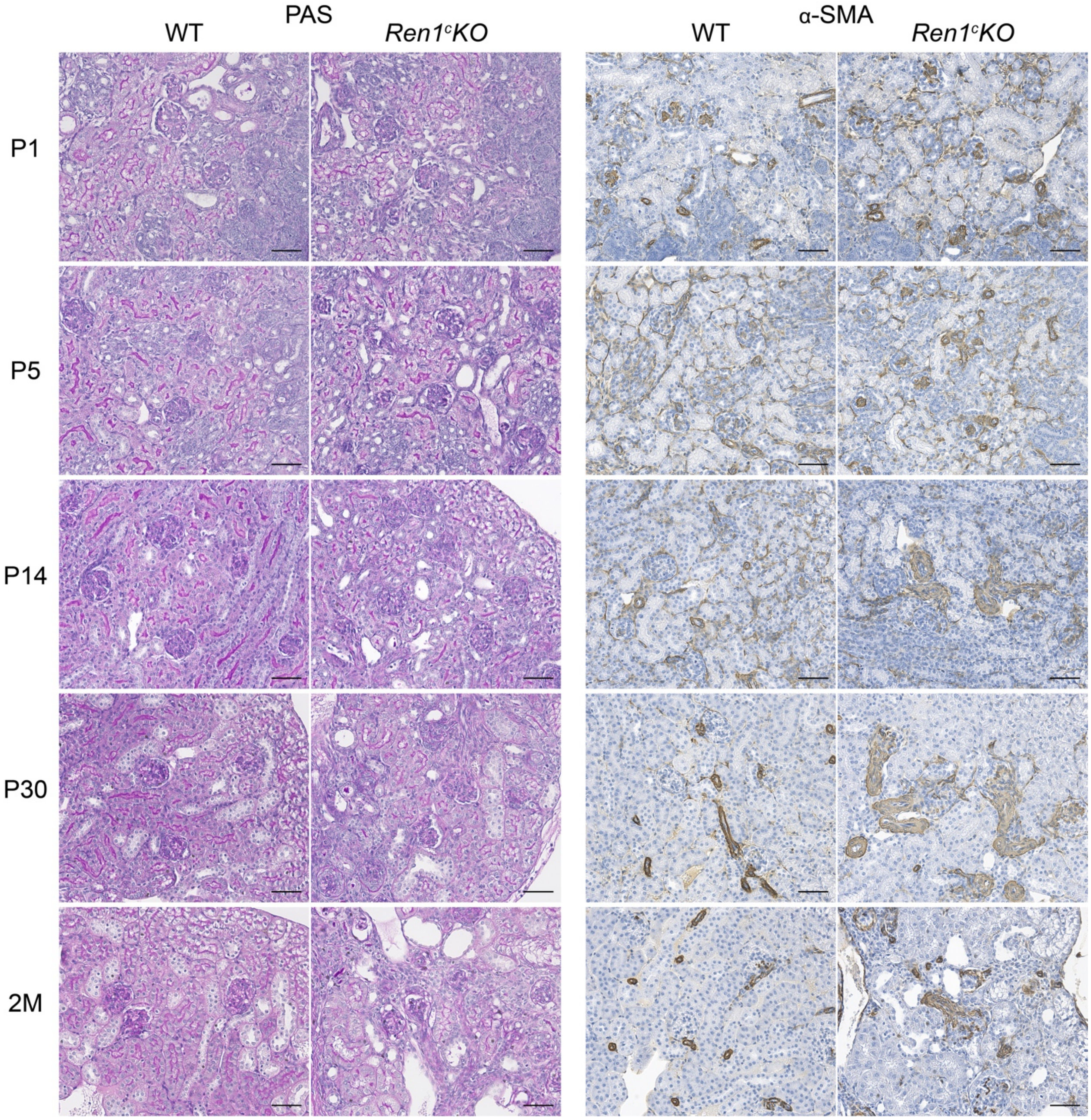

Supplemental Figure 3. Vascular abnormality in *Ren1^cKO* mice at different time points. PAS staining and immunohistochemistry for α-SMA in *Ren1^cKO* mouse kidneys and their controls at P1, P5, P14, P30, and 2 months of age. The afferent arteriolar hypertrophy was significantly detected in *Ren1^cKO* mice starting at P14. Scale bars, 50 μm. α-SMA; α-smooth muscle actin, *Ren1^cKO*; *Ren1^c* gene knockout, WT; wild-type.

Supplemental Figure 4

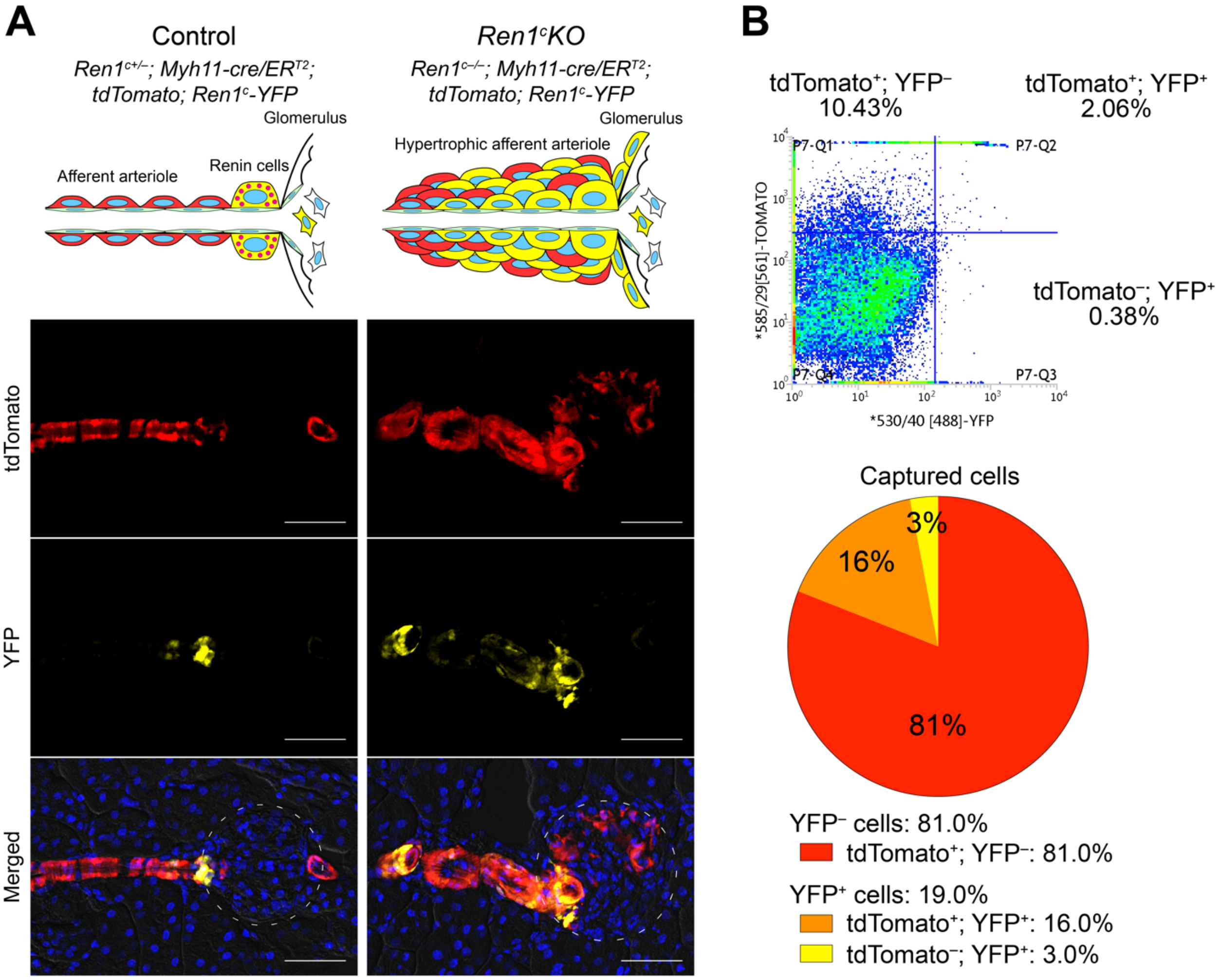

Supplemental Figure 4. Labeling *$Renin^{null}$* cells and other vascular SMCs. (**A**) Kidney sections from *$Ren1^{c+/–}$;Myh11-CreER^{T2};tdTomato;Ren1^{c}-YFP* mouse and *$Ren1^{c–/–}$;Myh11-CreER^{T2};tdTomato;Ren1^{c}-YFP* mouse. Vascular SMCs were labeled with tdTomato, and WT renin cells and *$Renin^{null}$* cells were labeled with YFP. The merged pictures also show nuclear staining by Hoechst and differential interference contrast microscopy. Scale bars, 50 μm. (**B**) FACS results of *$Ren1^{c–/–}$;Myh11-CreER^{T2};tdTomato;Ren1^{c}-YFP* mouse kidney. Of the isolated cells, 19% were the *$Renin^{null}$* cells (16.0% and 3.0% were tdTomato^{+};YFP^{+} and were tdTomato$^{–}$;YFP^{+}, respectively), and 81.0% were other tdTomato^{+};YFP$^{–}$ vascular SMCs. FACS; fluorescence-activated cell sorting, *$Ren1^{c}$KO*; *$Ren1^{c}$* gene knockout, SMCs; smooth muscle cells.

Supplemental Figure 5

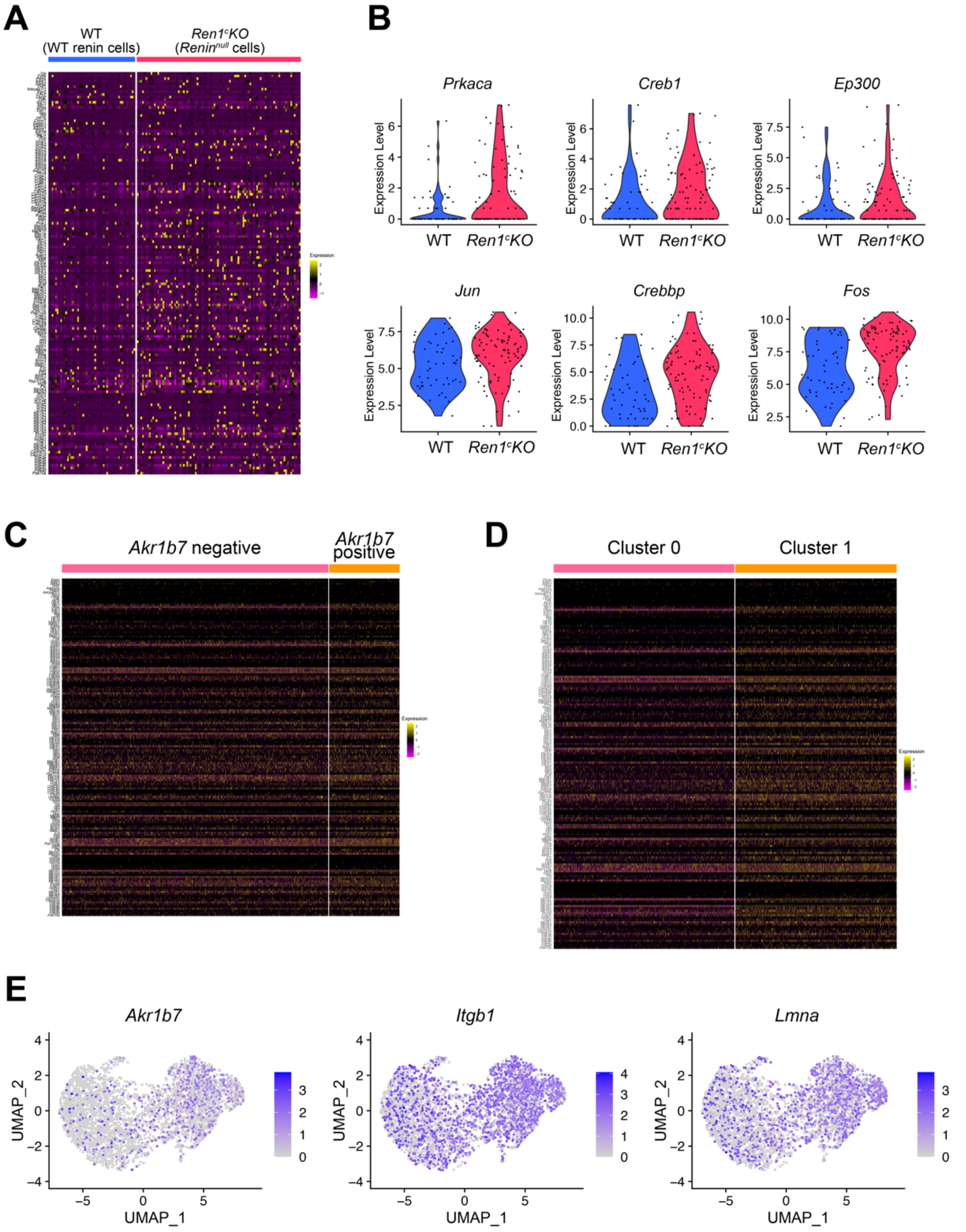

Supplemental Figure 5. scRNA-seq of cAMP pathway and mechanotransduction related genes. (**A**) Heatmap of the cAMP pathway genes from scRNA-seq of WT renin cells and *Renin^{null}* cells (C1 system). (**B**) Violin plots of the major cAMP pathway-related genes by C1 scRNAseq. (**C, D**) Heatmap of the cAMP pathway genes from scRNA-seq of *Ren1^{c}KO;SMMHC-CreER^{T2};tdTomato;Ren1^{c}-YFP* mice (Chromium System). (**E**) Feature plots of UMAP for *Akr1b7, Itgb1,* and *Lmna* in scRNA-seq of Chromium System. *Ren1^{c}KO*; *Ren1^{c}* gene knockout, scRNA-seq; single-cell-RNA sequencing, UMAP; Uniform Manifold Approximation and Projection, WT; wild-type.

Supplemental Figure 6

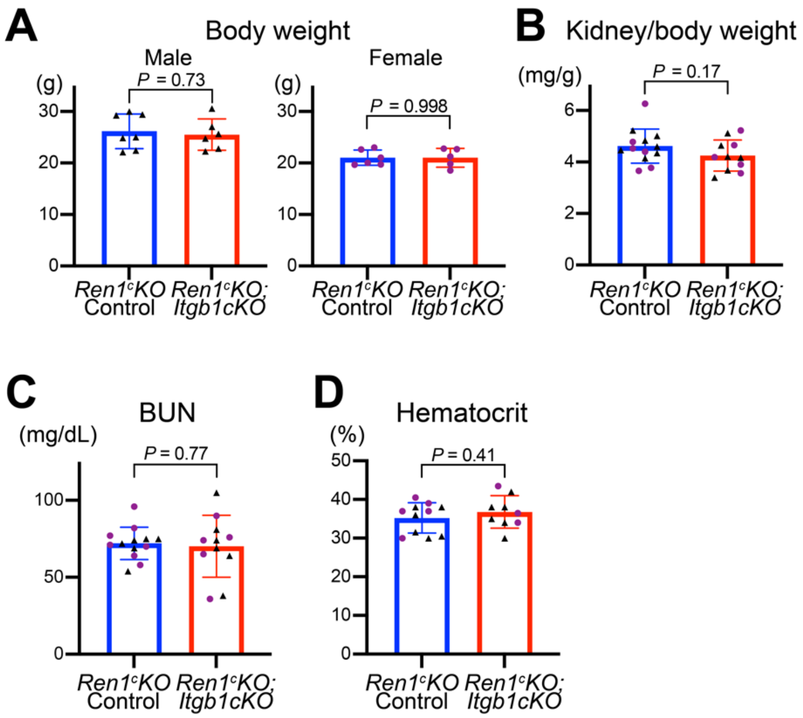

Supplemental Figure 6. The phenotype of the *Ren1^{c}KO;Itgb1cKO* mouse. (**A, B**) Body sizes were not different between *Ren1^{c}KO* control and *Ren1^{c}KO;Itgb1cKO* mice. There was no significant difference in body weight (**A**; male $n \geq 6$, female $n \geq 5$) and kidney/body weight rate (**B**; $n \geq 11$) (Student's t-test). (**C, D**) Blood tests did not differ in *Ren1^{c}KO* control and *Ren1^{c}KO;Itgb1cKO* mice. There was no significant difference in BUN (**C**; $n \geq 11$) and hematocrit (**D**; $n \geq 9$) (Student's t-test). All data are reported as means ± standard deviation. Black triangles show male samples, and purple dots show female samples. BUN; blood urea nitrogen, *Itgb1cKO*; deletion of the *Itgb1* gene in cells of the renin lineage, *Ren1^{c}KO*; *Ren1^c* gene knockout.

Supplemental Figure 7

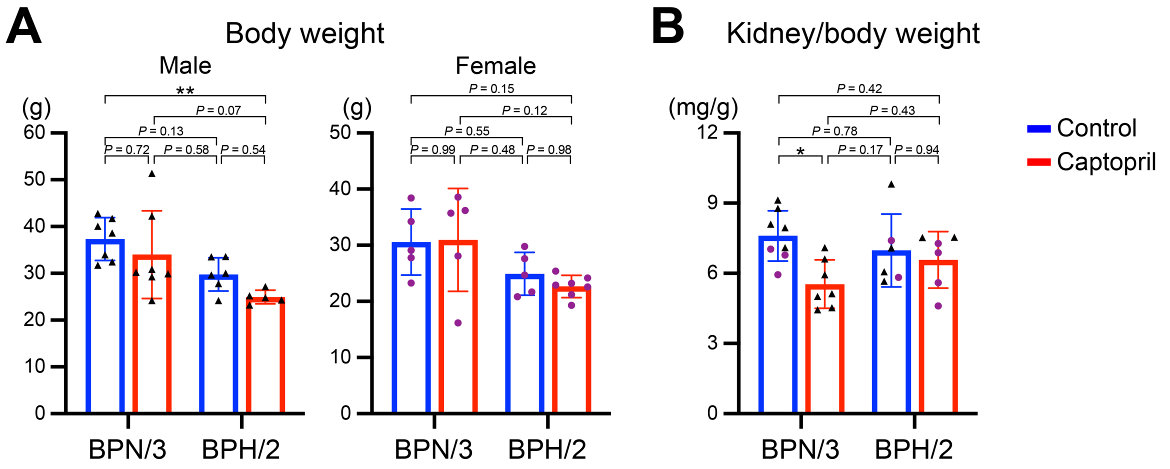

Supplemental Figure 7. Phenotypes of the BPN/3 and BPH/2 mice with and without long-term inhibition of RAS. (**A**) Body sizes were not different between with and without long-term usage of captopril in both BPN/3 and BPH/2 mice (*n*≥5, two-way ANOVA followed by Tukey's multiple comparison test). (**B**) Kidney/body weight rates were decreased by long-term usage of captopril in BPN/3 mice, but not in BPH/2 mice *(n*≥6, two-way ANOVA followed by Tukey's multiple comparison test). All data are reported as means ± standard deviation. Black triangles show male samples, and purple dots show female samples. **P*<0.05, ***P*<0.01.

Supplemental Figure 8

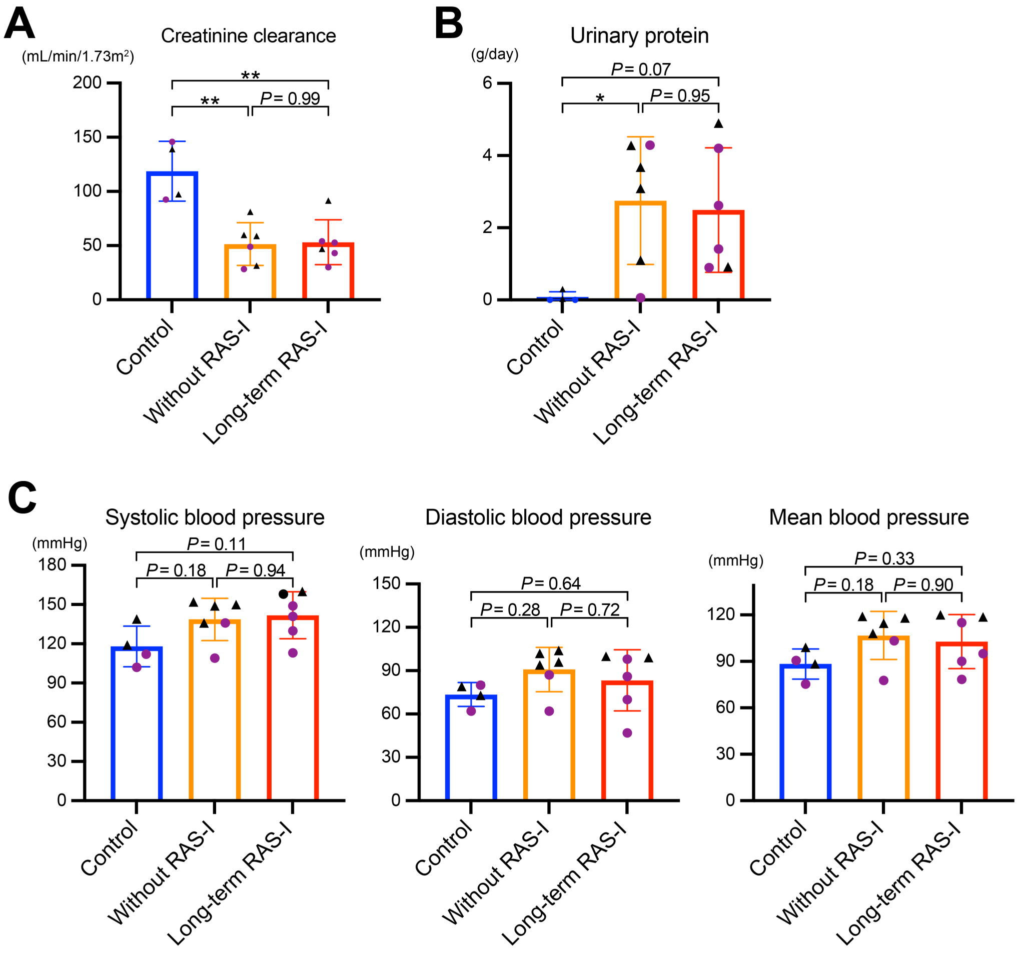

Supplemental Figure 8. The background of the human samples. (**A**) The creatinine clearance of patients with long-term usage of RASi (*n*=6) was not significantly different from patients with nephrosclerosis without RASi (*n*=6) (One-way ANOVA followed by Tukey's multiple comparisons test). (**B**) The urinary protein of patients with long-term usage of RASi (*n*=6) was not significantly different from patients with nephrosclerosis without RASi (*n*=6) (One-way ANOVA followed by Tukey's multiple comparisons test). (**C**) Blood pressure was not different between patients with long-term usage of RASi (*n*=6), healthy controls (*n*=4), and patients with nephrosclerosis without RASi (*n*=6) (One-way ANOVA followed by Tukey's multiple comparisons test). All data are reported as means ± standard deviation. Black triangles show male samples, and purple dots show female samples. *P<0.05, **P<0.01. RASi; renin-angiotensin system inhibitor.
